# GDNF Overexpression from the Native Locus Reveals its Role in the Nigrostriatal Dopaminergic System Function

**DOI:** 10.1371/journal.pgen.1005710

**Published:** 2015-12-17

**Authors:** Anmol Kumar, Jaakko Kopra, Kärt Varendi, Lauriina L. Porokuokka, Anne Panhelainen, Satu Kuure, Pepin Marshall, Nina Karalija, Mari-Anne Härma, Carolina Vilenius, Kersti Lilleväli, Triin Tekko, Jelena Mijatovic, Nita Pulkkinen, Madis Jakobson, Maili Jakobson, Roxana Ola, Erik Palm, Maria Lindahl, Ingrid Strömberg, Vootele Võikar, T. Petteri Piepponen, Mart Saarma, Jaan-Olle Andressoo

**Affiliations:** 1 Institute of Biotechnology, University of Helsinki, Helsinki, Finland; 2 Division of Pharmacology and Pharmacotherapy, Faculty of Pharmacy, University of Helsinki, Helsinki, Finland; 3 Department of Histology and Cell Biology, Umeå University, Umeå, Sweden; 4 Department of Physiology, Institute of Biomedicine and Translational Medicine, University of Tartu, Tartu, Estonia; 5 Department of Biochemistry and Developmental Biology, Institute of Biomedicine, University of Helsinki, Helsinki, Finland; 6 Neuroscience Center and Department of Biological and Environmental Sciences, University of Helsinki, Helsinki, Finland; Florey Institute of Neuroscience and Mental Health, AUSTRALIA

## Abstract

Degeneration of nigrostriatal dopaminergic system is the principal lesion in Parkinson’s disease. Because glial cell line-derived neurotrophic factor (GDNF) promotes survival of dopamine neurons *in vitro* and *in vivo*, intracranial delivery of GDNF has been attempted for Parkinson’s disease treatment but with variable success. For improving GDNF-based therapies, knowledge on physiological role of endogenous GDNF at the sites of its expression is important. However, due to limitations of existing genetic model systems, such knowledge is scarce. Here, we report that prevention of transcription of *Gdnf* 3’UTR in *Gdnf* endogenous locus yields GDNF hypermorphic mice with increased, but spatially unchanged GDNF expression, enabling analysis of postnatal GDNF function. We found that increased level of GDNF in the central nervous system increases the number of adult dopamine neurons in the substantia nigra pars compacta and the number of dopaminergic terminals in the dorsal striatum. At the functional level, GDNF levels increased striatal tissue dopamine levels and augmented striatal dopamine release and re-uptake. In a proteasome inhibitor lactacystin-induced model of Parkinson’s disease GDNF hypermorphic mice were protected from the reduction in striatal dopamine and failure of dopaminergic system function. Importantly, adverse phenotypic effects associated with spatially unregulated GDNF applications were not observed. Enhanced GDNF levels up-regulated striatal dopamine transporter activity by at least five fold resulting in enhanced susceptibility to 6-OHDA, a toxin transported into dopamine neurons by DAT. Further, we report how GDNF levels regulate kidney development and identify microRNAs miR-9, miR-96, miR-133, and miR-146a as negative regulators of GDNF expression via interaction with *Gdnf* 3’UTR *in vitro*. Our results reveal the role of GDNF in nigrostriatal dopamine system postnatal development and adult function, and highlight the importance of correct spatial expression of GDNF. Furthermore, our results suggest that 3’UTR targeting may constitute a useful tool in analyzing gene function.

## Author Summary

Intracranial delivery of GDNF has been attempted for Parkinson’s disease (PD) treatment but with variable success. For improving GDNF-based therapies, knowledge on physiological role of endogenous GDNF at the sites of its expression is important. However, due to limitations of existing genetic model systems, such knowledge is scarce. Here, we utilize an innovative genetic approach by targeting the 3’UTR regulation of *Gdnf* in mice. Such animals express elevated levels of *Gdnf* exclusively in natively *Gdnf*-expressing cells, enabling dissection of endogenous GDNF functions *in vivo*. We show that endogenous GDNF regulates dopamine system development and function and protects mice in a rodent PD model without side effects associated with ectopic GDNF applications. Further, we report how GDNF levels regulate kidney development and identify microRNAs which control GDNF expression. Our study highlights the importance of correct spatial expression of GDNF and opens a novel approach to study gene function in mice.

## Introduction

Exogenously applied glial cell line-derived neurotrophic factor (GDNF) promotes the survival, function, and neurite growth of nigrostriatal dopamine (DA) neurons both *in vitro* and *in vivo* [1,2]. The classic motor deficit in Parkinson’s disease is characterized by a gradual loss of nigrostriatal DA neurons, leading to a reduction in striatal dopamine levels, resting tremor, rigidity, and an inability to initiate voluntary movement [3]. Intracranial delivery of GDNF has been tested in clinical trials for treating Parkinson’s disease (PD); however, both the efficacy and the side effects of this treatment vary widely [3–6]. Increasing the therapeutic efficacy of GDNF requires a better understanding of its physiological role; however, our knowledge regarding the postnatal role of GDNF is currently limited. Knockout mice that lack *Gdnf* or its receptors (*Gfrα1* and *Ret*) die at birth due to a lack of kidneys, but with intact nigrostriatal DA system which undergoes developmental maturation during the first three post-natal weeks [7,8]. It has been reported that 50% reduction in GDNF levels in adult *Gdnf* conditional knock-out mice has profound consequences on midbrain dopamine neuron survival upon aging [9]. However, our recent study with *Gdnf* conditional knock-out mice utilizing three Cre systems including the repetition of the experiments performed in [9] did not reveal loss of DA neurons after GDNF deletion or reduction at any age [10]. Based on current evidence it is possible that GDNF either has no physiological role in the brain DA system, that GDNF reduction or deletion in the brain is compensated by another mechanism, or that GDNF regulates the DA system at the functional level, rather than at the level of supporting the survival of the DA cell bodies in the midbrain. Moreover, although GDNF is known to be essential for initiating kidney development [7], our understanding of the role of endogenous GDNF in kidney maturation has remained limited.

Here, we report generation and analysis of mice carrying *Gdnf* hypermorphic (*Gdnf* ^*hyper*^) allele, generated by insertion of a cassette containing bovine growth hormone polyA signal after the stop-codon in *Gdnf* endogenous locus preventing transcription into *Gdnf* wild type 3’UTR. These mice have increased–but spatially unchanged–expression of the endogenous *Gdnf* gene. While *Gdnf* ^*hyper/hyper*^ mice die by postnatal day 18 (P18) due to kidney defects, *Gdnf* ^*wt/hyper*^ mice are healthy and only display mild occasional reduction in kidney size. *Gdnf* ^*wt/hyper*^ animals revealed that GDNF has an important role in the postnatal nigrostriatal system development and adult function and clarified which aspects of the nigrostriatal dopaminergic system structure and function are regulated by GDNF. They also enabled analysis of GDNF function in kidney maturation beyond the first steps in renal development.

## Results

### Generation of *Gdnf* hypermorphic allele and *in vitro* analysis

**Fig 1:**
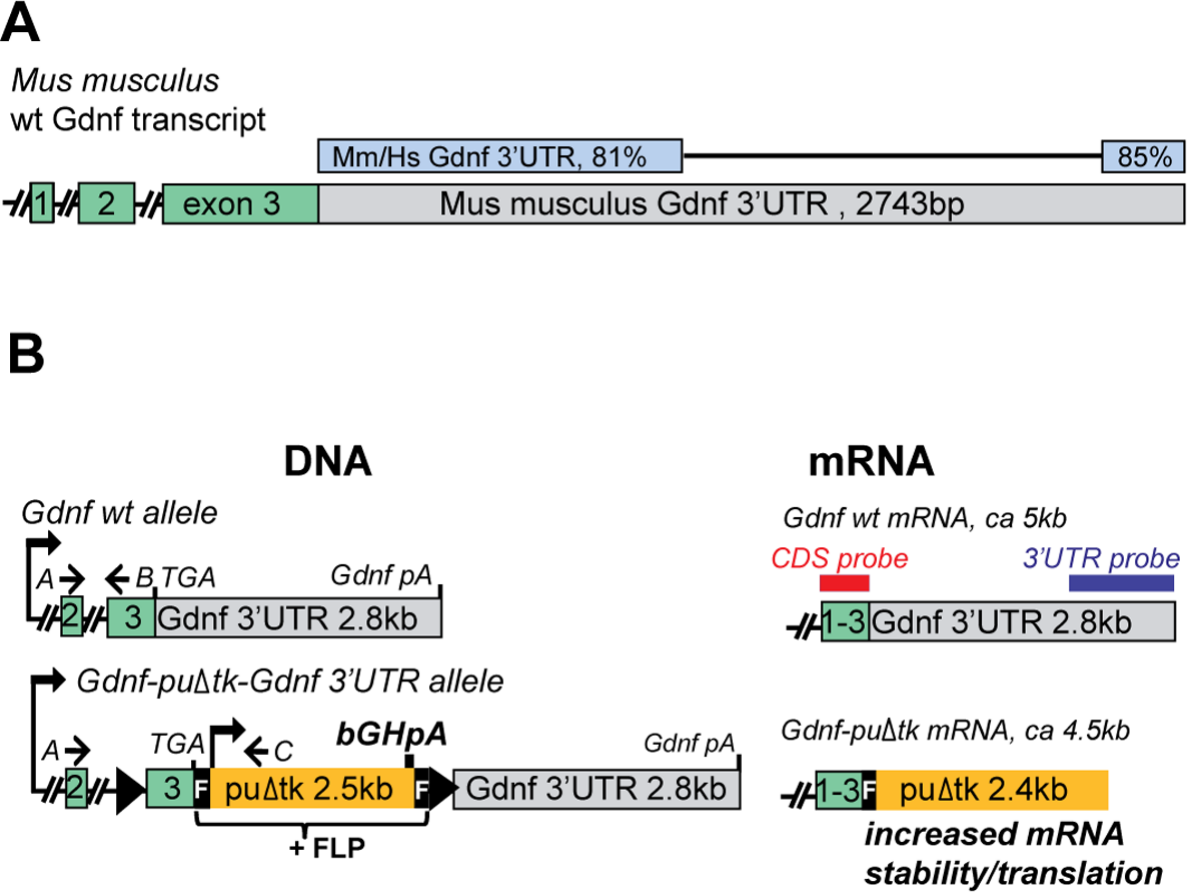
*Gdnf* 3’UTR and generation of GDNF hypermorphic allele. **(A)** The 3’UTR of the *Gdnf* mRNA is evolutionarily conserved. Exons and the 3’UTR are drawn to scale. Percent of identity between the human (Hs) and mouse (Mm) sequences is indicated. Source: Blast. **(B)** Schematic representation of the *Gdnf* hypermorphic allele. *A* and *C* designate the primers used for *Gdnf* mRNA sequence analysis in targeted mice (see S2F and S2G Fig); primers *A* and *B* were used for *Gdnf* QPCR. The bGHpA, puΔtk, loxP sites (black triangles), and F-FRT sites are indicated. The blue and red bars indicate the probes used for *in situ* hybridization.

In the process of generating a conditional knockout (or “floxed”) *Gdnf* allele [10], we noted that the 3’UTR of *Gdnf* is relatively long and evolutionarily conserved (Fig 1A). Since *Gdnf* 3’UTR inhibits reporter gene expression in a cell line [11] we decided to analyze *Gdnf* 3’UTR function *in vivo* by insertion of an FRT-flanked *puΔtk* cassette [12] after the stop codon in the *Gdnf* locus in embryonic stem (ES) cells. The *puΔtk* cassette contains the bovine growth hormone polyadenylation (bGHpA) signal, which induces termination of transcription and is commonly used in gene-trap experiments in mice (Fig 1B). We used a luciferase-based reporter assay to confirm that the bGHpA signal prevents transcription into the *Gdnf* 3’UTR in our construct (S1A and S1B Fig) and yields correctly sized fusion mRNA (S1C Fig). Using a reporter gene assay, we found an 8-fold increase in luciferase expression from the construct containing *Firefly-puΔtk* proceeded by *Gdnf* 3’UTR (relative to *Firefly-Gdnf* 3’UTR) in a cell line derived from human embryonic kidney cells (HEK293) and a 2-fold increase in a cell line derived from human brain cells (U87) (S1D Fig). We also observed similar inhibitory effects on reporter gene expression, regardless of whether the *Gdnf* 3’UTR was cloned downstream of a sea pansy (*Renilla reniformis*) or Photinini firefly (*Photinus pyralis*) luciferase in both cell lines (S1E Fig), suggesting that the inhibition of expression by *Gdnf* 3’UTR is not limited to one cell type or dependent on the preceding gene. Blocking transcription with actinomycin D revealed that the *Firefly-puΔtk* yields a more stable gene product than *Firefly-Gdnf* 3’UTR, suggesting that negative regulation via *Gdnf* 3’UTR occurs at the post-transcriptional level (S1F Fig).

Next, we generated mice carrying the *Gdnf-FRT-puΔtk*-FRT-*Gdnf* 3’UTR allele (S1G–S1I Fig). Based on the above experiments and a previously published study [11] the allele was hypothesized to result in elevated expression of endogenous *Gdnf* (Fig 1B, S1D–S1F Fig). Homozygous *Gdnf* ^*puΔtk/puΔtk*^ mice died before P18 due to extremely small, morphologically disorganized kidneys (see below). In contrast, kidney defects were mild (or absent) in heterozygous mice (S1 Table), which were born at the expected Mendelian frequency (S2 Table) and appeared otherwise phenotypically normal (see below).

### Analysis of *Gdnf* mRNA expression levels and sites in peripheral tissues of *Gdnf* ^*puΔtk*^ mice

**Fig 2:**
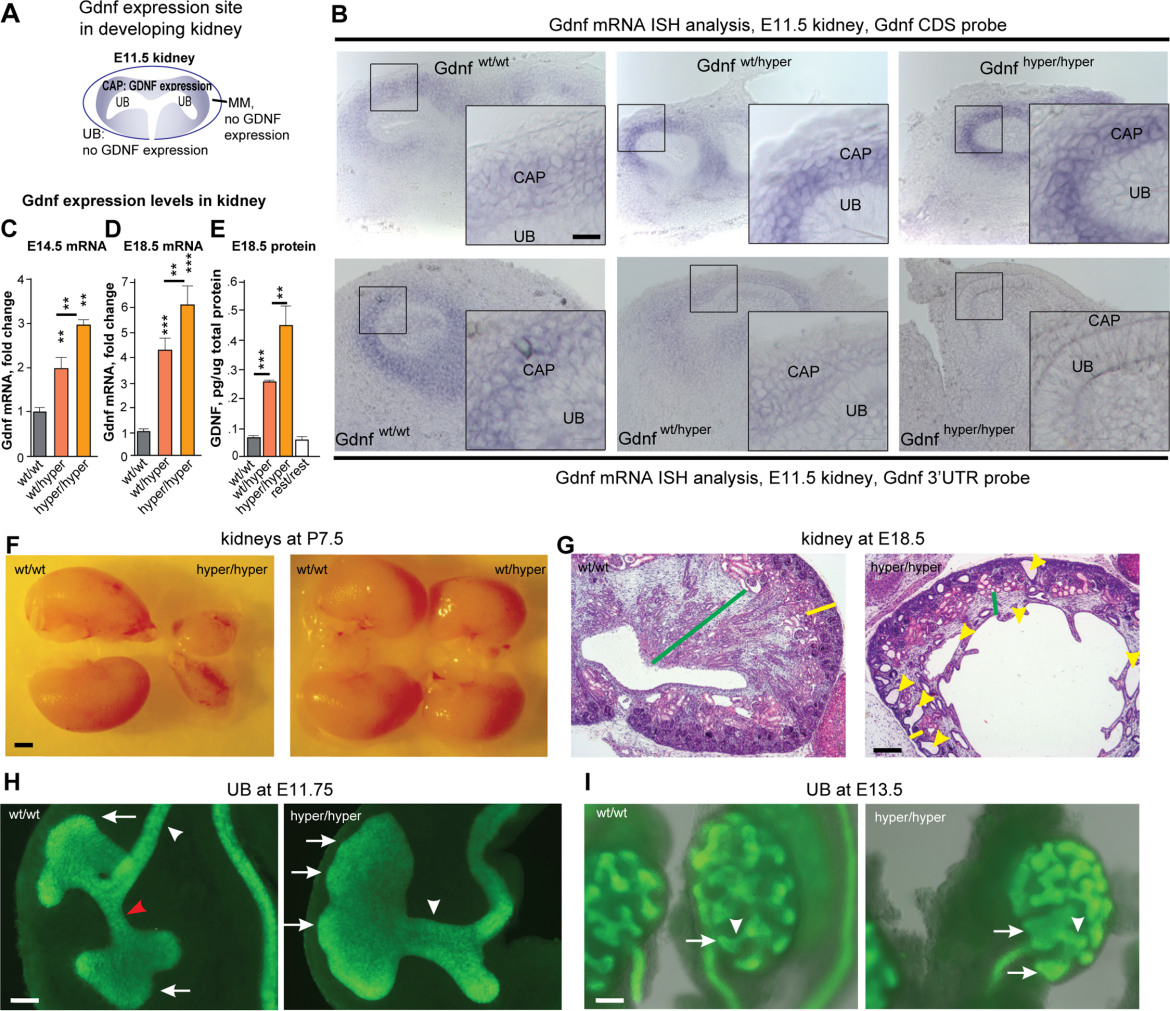
*Gdnf* levels are a critical determinant of embryonic renal growth and morphogenesis. **(A)** Schematic representation of *Gdnf* expression (blue) in the mouse kidney at E11.5. **(B)** Representative image of *in situ* hybridization of *Gdnf* mRNA (blue) in the urogenital tract of E11.5 mice. N = 4 mice/group. **(C-E)** *Gdnf* mRNA [E14.5 (C) and E18.5 (D)] and protein [E18.5; (E)] levels in the kidney measured using QPCR (C and D) and ELISA (E). N = 2–10 mice/group. **(F)** Representative image of kidneys obtained from P7.5 mice. **(G)** Representative image of hematoxylin-and-eosin‒stained sections from E18.5 kidneys. The renal cortex is indicated with a yellow bar, the medulla is indicated with a green bar, and collecting duct cysts are indicated with yellow arrowheads. **(H)** At the time of renal differentiation initiation (E11.75), a wild-type kidney (left) contains a typical UB branching pattern with an interim stalk (red arrowhead), elongated ureter stalk (white arrowhead), and locally enlarged UB tips (arrows). In contrast, a kidney from a *Gdnf* ^*hyper/hyper*^ embryo (right) contains one large UB that appears bumpy (arrows), lacks an interim stalk, and lacks normal elongation of the UB (white arrowhead). **(I)** Images of a wild-type (left) and *Gdnf* ^*hyper/hyper*^ (right) kidney at E13.5; the kidney from the *Gdnf* ^*hyper/hyper*^ embryo is smaller in size, has enlarged ureteric buds (arrows), and shortened stalks (arrow head). For F-I, N = 3–20 mice/group. Scale bars: B, 10 μm; F, 1 mm; G, 300 μm H, 50 μm; I, 100 μm. Abbreviations: E, embryonic day; MM, metanephric mesenchyme; P, postnatal day; UB, ureteric bud. In this and subsequent figures, all summary data are presented as the mean ± SEM; *P<0.05, **P<0.01, and ***P<0.001; Student’s *t*-test, unless noted otherwise.

We first measured the location and levels of *Gdnf* expression in the developing kidney, testis and developing hind limb where the expression sites of *Gdnf* are well established [13,14], http://developingmouse.brain-map.org. Because antibodies that selectively and specifically bind endogenous GDNF protein in histology sections are not currently available, we used *in situ* hybridization to detect *Gdnf* mRNA. During embryonic kidney development, the expression of *Gdnf* is limited to a structure of metanephric mesenchyme called cap condensate, which surrounds a *Gdnf*-negative epithelial ureteric bud (Fig 2A). A probe spanning the *Gdnf*-coding sequence (comprising exons 1 through 3) revealed a *Gdnf* ^*puΔtk*^ allelic dose-dependent increase in *Gdnf* mRNA levels, but no difference in the site of expression between wild-type, heterozygous, and homozygous mice (Fig 2B, upper panel). Thus we designated the *Gdnf* ^*puΔtk*^ allele as *Gdnf* hypermorphic or *Gdnf* ^*hyper*^ allele. Further analysis revealed that a probe complementary to 525 bp of the 3’ end of the *Gdnf* 3’UTR revealed fewer *Gdnf* 3’UTR-containing transcripts in *Gdnf* ^*wt/hyper*^ mice compared to *Gdnf* ^*w/wt*^ mice. As expected, no signal was detected in *Gdnf* ^*hyper/hyper*^ mice using this probe (Fig 2B, lower panel), suggesting that transcription of *Gdnf* wt 3’UTR is prevented by the puΔtk cassette, as it did in cell line (S1B Fig). A similar increase in *Gdnf* expression was measured in Sertoli cells (in the testes) and in the developing hind limbs (S2A and S2B Fig). Quantification of *Gdnf* mRNA and GDNF protein levels in the developing kidneys and testes also revealed a *Gdnf* ^*hyper*^ allelic dose-dependent increase in both organs (Fig 2C–2E, S2C and S2D Fig), and removal of the puΔtk cassette by crossing with Deleter-FLP mice (Fig 1B; “*Gdnf 3’UTR* ^*rest/rest*^ mice”) restored GDNF expression to wild-type levels (Fig 2E, S2C and S2D Fig). Thus, we concluded that the *Gdnf* ^*hyper*^ allele indeed resulted in elevated expression of GDNF in peripheral tissues.

To further analyze the *Gdnf* transcript in *Gdnf* ^*hyper*^ mice we applied Northern blotting to analyze *Gdnf* mRNA in testis. We observed a major band at the expected size of the predicted *Gdnf*-puΔtk fusion transcript (S2E Fig). We also sequenced the *Gdnf* transcripts from testis in *Gdnf* ^*wt/wt*^, *Gdnf* ^*hyper/hyper*^, and *Gdnf 3’UTR* ^*rest/rest*^ mice using RT-PCR. We used a forward primer spanning *Gdnf* exon 2 and a reverse primer spanning puΔtk (starting 428 bp downstream from the TGA stop codon of *Gdnf*) (S2F and S2G Fig); our analysis revealed a *Gdnf-puΔtk* fusion transcript with the predicted size and sequence (S2G Fig).

### Kidney size and morphology are negatively regulated by excess GDNF levels

Ureteric bud outgrowth–the first step in kidney development–is induced by GDNF [7]. Thus, homozygous *Gdnf*-knockout mice lack kidneys, and 20–30% of heterozygous GDNF-knockout mice have only one kidney [15]. *In vitro*, exogenous GDNF promotes the growth of ectopic ureteric buds in embryonic tissue explants and expansion of endogenous ureteric buds tips [16]. However, how endogenous GDNF regulates subsequent steps in kidney development *in vivo* is poorly understood. *In vitro* GDNF protein application studies [16] and results from the *Gdnf* knockout mice [15] suggest that the overexpression of *Gdnf* in our hypermorphic mice might cause enlargement of the kidneys. In contrast, we found that overexpression of GDNF negatively regulates kidney size and morphological maturation (Fig 2F and 2G; S1 Table) in a concentration-dependent manner (Fig 2B–2E). An analysis of the early steps in kidney development (i.e., in E11.5 through E11.75) in *Gdnf* ^*hyper/hyper*^ mice revealed that excess GDNF (Fig 2B, upper panel) induces hypertrophic ureteric bud formation and impairs the development and elongation of the stalk (Fig 2H). At mid-gestation (i.e., E13.5), these enlarged ureteric buds and absent or shortened stalks are still observed, and the initial signs of reduced kidney size emerge (Fig 2I). During late embryogenesis, the kidneys in *Gdnf* ^*hyper/hyper*^ mice fail to reach normal size, resulting in severe renal hypodysplasia with disorganized medulla-cortex compartmentalization, reduced cortical and medullar areas, and cysts in the collecting ducts (Fig 2G). An analysis of kidney function by measuring serum electrolytes revealed that the kidneys of *Gdnf* ^*hyper/hyper*^ mice function poorly (S2H and S2I Fig). However, in *Gdnf* ^*wt/hyper*^ mice the kidney function remains relatively normal (S2H–S2K Fig). Finally, renal development was restored by crossing with Deleter-FLP mice (*Gdnf 3’UTR* ^*rest/rest*^; S2L Fig). These data indicate that in kidney development, the correct GDNF levels are important as both lack of GDNF and excess GDNF result in failure of kidney development and function. Importantly, in *Gdnf* ^*wt/hyper*^ mouse the kidneys were functional and mice were healthy, providing us a unique animal model to study the postnatal function of GDNF in the CNS.

### Analysis of *Gdnf* mRNA expression levels and sites in the CNS of *Gdnf* ^*hyper*^ mice

**Fig 3:**
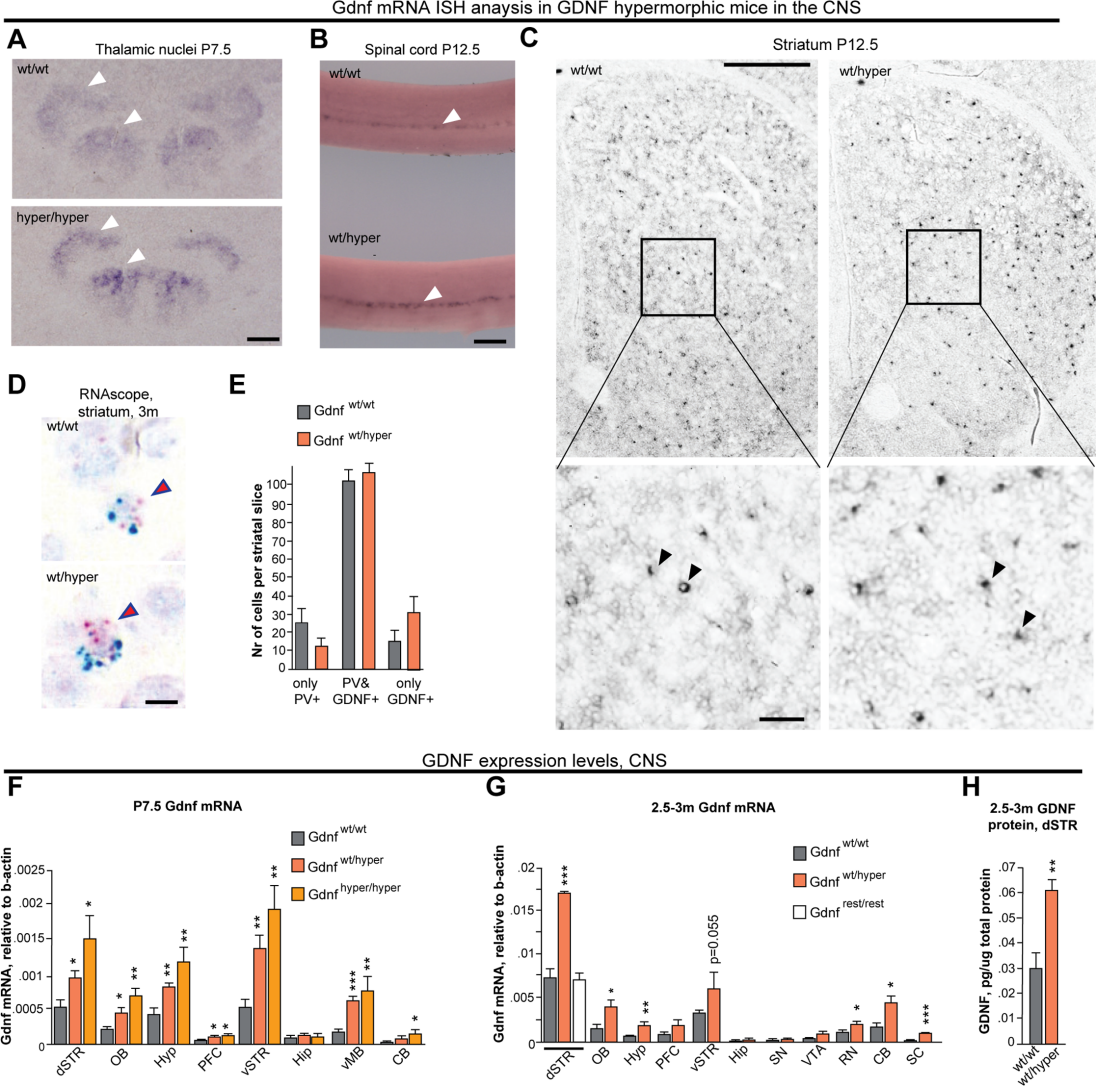
*Gdnf* expression is increased in cells that normally express *Gdnf*. **(A)** *In situ* hybridization showing *Gdnf* mRNA expression in thalamic nuclei at P7.5 (white arrowheads). **(B)** *In situ* hybridization showing *Gdnf* mRNA expression in Clarke’s column in the thoracic part of the spinal cord at P12.5 (white arrowheads). **(C)** *Gdnf* mRNA-positive cells (black dots) in the whole striatum (upper panel); a magnified view of *Gdnf* mRNA-positive cells (arrowheads) is shown in the lower panel. **(D)** Representative images of *Pvalb (PV*, blue) and *Gdnf* (red) mRNA in the striatum of 3-month-old mice detected using RNAscope. **(E)** Summary of *PV*-positive only, *Gdnf*-positive only, and double-positive cells in striatal slices obtained from *Gdnf* ^*wt/wt*^ and *Gdnf* ^*wt/hyper*^ mice; N = 5 animals/group. **(F)** QPCR analysis of *Gdnf* mRNA levels in the indicated brain regions in P7.5 mice; N = 6–8 mice/group. **(G)** QPCR analysis of *Gdnf* mRNA levels in the indicated brain regions of adult mice; N = 4–8 mice/group. **(H)** ELISA analysis of GDNF protein levels in the dorsal striatum of adult mice; N = 6–8 mice/group. Scale bars: A, 150 μm; B, 200 μm; C (upper panels), 500 μm; C (lower panels), 50 μm; D, 10 μm. Abbreviations: dSTR, dorsal striatum; OB, olfactory bulb; Hyp, hypothalamus; PFC, prefrontal cortex; vSTR, ventral striatum; Hip, hippocampus; vMB, ventral midbrain; CB, cerebellum; RN, dorsal raphe nucleus; SC, spinal cord; SN, substantia nigra; VTA, ventral tegmental area; PV, parvalbumin; m, months; P, postnatal day.

Next, we set to analyze the site of *Gdnf* expression in the CNS of GDNF hypermorphic mice. We utilized *in situ* hybridization analysis of *Gdnf* mRNA in thalamic nuclei and spinal cord and observed identical spatial expression patterns between genotypes (Fig 3A and 3B). In the striatum, where *Gdnf* mRNA levels peak at P12.5 [17,18], the spatial pattern of *Gdnf* expression was limited to a discrete set of sparsely distributed cells, interspaced with *Gdnf* nonexpressing cells, consistent with previous reports [17,19] and similar between *Gdnf* ^*wt/hyper*^ and *Gdnf* ^*wt/wt*^ mice (Fig 3C). In the striatum, *Gdnf* is expressed primarily by *parvalbumin* (*Pvalb*)—expressing inhibitory neurons [17]. We therefore used RNAscope, a novel high-sensitivity *in situ* hybridization method for double-staining *Pvalb* and *Gdnf* mRNAs in histological sections [20]. First, we verified the specificity of RNAscope probes for *Pvalb* and *Gdnf* mRNA in the adult cerebellum and E14.5 kidney (S2M Fig), where the expression of *Pvalb* and *Gdnf*, respectively, has been well characterized. *Pvalb/Gdnf* double-staining of the striatum of adult mice confirmed that the expression of *Gdnf* in *Gdnf* ^*wt/hyper*^ mice is largely retained to *Pvalb*-expressing cells, similar to wild-type controls (Fig 3D and 3E).

To assess the level of *Gdnf* mRNA in several CNS regions including dorsal striatum, olfactory bulb, hypothalamus, ventral striatum, ventral midbrain and cerebellum in P7.5 *Gdnf* ^*wt/hyper*^ and *Gdnf* ^*hyper/hyper*^ animals and in 2.5–3 month old adult *Gdnf* ^*wt/hyper*^ mice we utilized quantitative RT-PCR. We observed an increase of *Gdnf* mRNA in *Gdnf* ^*wt/hyper*^ and *Gdnf* ^*hyper/hyper*^ mice in comparison to wild-type littermate controls (Fig 3F and 3G). To examine whether the increase in *Gdnf* mRNA level is reflected in the GDNF protein levels we used ELISA assay and noted that GDNF protein levels were increased by approximately two fold in the dorsal striatum of adult *Gdnf* ^*wt/hyper*^ mice (Fig 3H). Notably, we observed no elevation in *Gdnf* mRNA levels in the substantia nigra of adult *Gdnf* ^*wt/hyper*^ mice (Fig 3G). We conclude that elevation in *Gdnf* mRNA expression in *Gdnf* ^*hyper*^ mice occurs in most brain structures which naturally express *Gdnf* in the brain, and that *Gdnf* mRNA and protein levels in the dorsal striatum of adult *Gdnf* ^*wt/hyper*^ mice are two fold increased.

### Increased GDNF levels increase the nigrostriatal dopaminergic system during development

**Fig 4:**
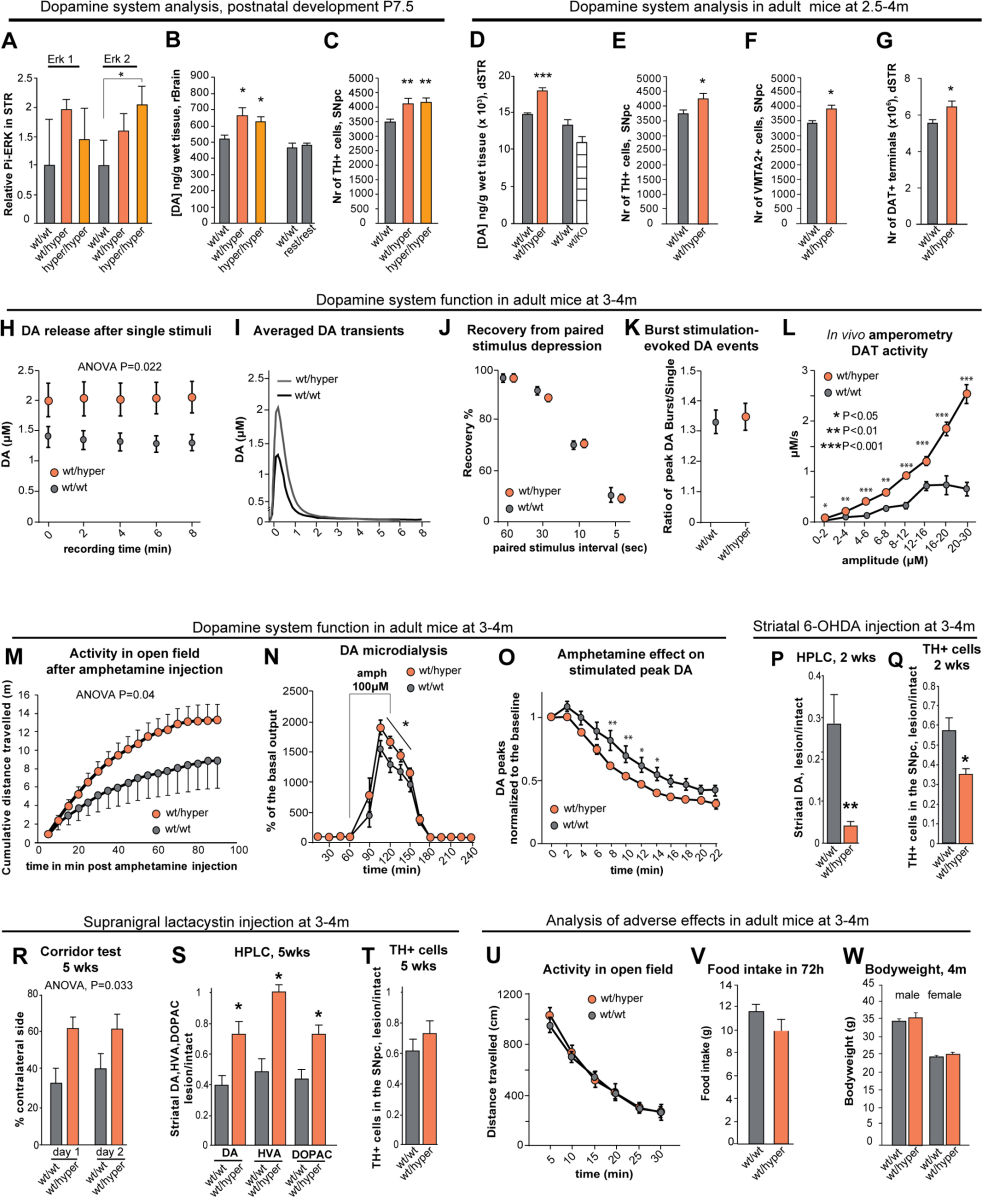
Increased endogenous GDNF expression affects the development and function of the nigrostriatal dopaminergic system. **(A)** Levels of phosphorylated ERK2 at P7.5 in the striatum of *Gdnf* ^*wt/wt*^, *Gdnf* ^*wt/hyper*^ and *Gdnf* ^*hyper/hyper*^ mice. N = 5 mice/group; ERK was used for normalization. **(B)** HPLC analysis of DA levels in the rostral brain; N = 5–8 mice/group (F = 7.44, P = 0.016). **(C)** Quantification of tyrosine hydroxylase (TH)-positive (a marker of DA neurons) cells in the SNpc; N = 6–8 mice/group (F = 7.44, P = 0.0048). **(D)** HPLC analysis of DA levels in the dSTR; N = 11 for *Gdnf* ^*wt/wt*^, 8 for *Gdnf* ^*wt/hyper*^ mice/group (P = 0.000164). HPLC analysis of DA levels in the dorsal striatum of *Gdnf 3’UTR* ^*wt/wt*^ and *Gdnf* ^*wt/KO*^ mice; N = 6 mice/group. **(E-F)** The number of TH-positive (E; N = 8 *Gdnf* ^wt/wt^, N = 7 *Gdnf* ^wt/hyper^; P = 0.025) and VMAT2-positive neurons (F; N = 7 *Gdnf* ^wt/wt^, N = 7 *Gdnf* ^wt/hyper^; P = 0.016) in the SNpc. **(G)** The number of DAT+ varicosities (N = 9 *Gdnf* ^wt/wt^, N = 7 *Gdnf* ^wt/hyper^; P = 0.042) in the dSTR. **(H-K)** Cyclic voltammetry analysis of acute striatal slices (see also S3J Fig); N = 5–7 mice/group with 1–3 slices per mouse. **(H)** DA release in response to electrical stimulation [two-way repeated measures ANOVA, F (1,29) = 5.866]; **(I)** Averaged traces of DA events. **(J)** Short-term depression of striatal DA release after prior DA exocytosis, shown as percent of the first DA release. **(K)** The ratio of DA release after a single stimulus and after a 5 pulse burst at 20Hz. **(L)** *In vivo* amperometry following intrastriatal DA injection reveals that dopamine transporter (DAT) activity in *Gdnf* ^*wt/hyper*^ mice is dependent on the concentration of DA; N = 4 mice/group (F = 47.931). **(M)** Locomotor activity after an injection of amphetamine (1 mg/kg, i.p.); N = 9–10 mice/group (F = 4.386, P = 0.04). **(N)** *In vivo* microdialysis analysis of extracellular striatal DA levels; amphetamine was applied as indicated by the horizontal bar; N = 9 mice/group. **(O)** Cyclic voltammetry analysis shows that amphetamine (5 μM) depletes stimulated DA release faster in the striata of *Gdnf* ^*wt/hyper*^ mice compared to *Gdnf* ^*wt/wt*^ mice; two-way repeated-measures ANOVA reveals an effect of time (P<0.0001) and genotype (P = 0.031), as well as an interaction between time and genotype (P = 0.049); N = 6 mice/group with 1–3 slices per mouse. **(P-Q)** Analysis of a 6-OHDA induced PD model. **(P)** Quantification of DA in the dSTR 2 weeks after striatal 6-OHDA injection, relative to the intact side (N = 12 *Gdnf* ^wt/wt^; N = 10 *Gdnf* ^wt/hyper^), (F = 40.62, P = 0.00549, Students t-test). The intact and lesioned side differed significantly (P = 2.71×10^−15^). **(Q)** Quantification of TH-positive neurons in the SNpc 2 weeks after striatal 6-OHDA injection, relative to the intact side, (F = 7.04, P = 0.0143, Students t-test). The intact and lesioned side differed significantly (P = 3.00×10^−11^). **(R-T)** Analysis of a lactacystin-induced PD model. **(R)** The percentage of sugar pellet retrievals from the contralateral side in the corridor test; N = 5–7 mice/group (F = 6.087, P = 0.033). **(S)** Quantification of DA, DOPAC, and HVA in the dSTR 5 weeks after supranigral lactacystin injection, relative to the intact side; N = 5 *Gdnf* ^*wt/wt*^, N = 7 *Gdnf* ^*wt/hyper*^; P = 0.046 for DA, P = 0.015 for DOPAC, P = 0.011 for HVA. The intact and lesioned side differed significantly; P = 0.00016 for DA, P = 0.015 for DOPAC, P = 0.010 for HVA. **(T)** Quantification of TH-positive neurons in the SNpc 5 weeks after lactacystin injection, relative to the intact side; N = 4 *Gdnf* ^*wt/wt*^, N = 7 *Gdnf* ^*wt/hyper*^; P = 0.236. The intact and lesioned side differed significantly (P = 0.00029). **(U-W)** Evaluation of side effects associated with intracranial ectopic GDNF expression. **(U)** Spontaneous locomotor activity in an open field; N = 31–34 mice/group. **(V)** Food intake by adult mice during a 72-hour period; N = 10 mice/group. **(W)** Body weight of adult mice; N = 9–34 mice/group. Abbreviations: DA, dopamine; DOPAC, 3,4-dihydroxyphenylacetic acid; HVA, homovanillic acid; dSTR, dorsal striatum; SNpc, substantia nigra pars compacta.

In the first three postnatal weeks, DA neurons experience several major developmental changes, including maturation of striatal target innervation and programmed cell death in the substantia nigra pars compacta [7,8]. The postnatal function of endogenous GDNF in the brain’s dopaminergic system is poorly understood [10]. To assess whether increased GDNF levels in the GDNF hypermorphic mice result in changes in nigrostriatal DA system during development, we first analyzed the striatal levels of phosphorylated extracellular signal-regulated kinase (Erk) a known target in GDNF signaling [7] using western blotting. We found that the levels of phosphorylated ERK2 were increased in the striatum of *Gdnf* ^*wt/hyper*^ and *Gdnf* ^*hyper/hyper*^ mice at P7.5 (Fig 4A), indicating increased GDNF signaling. Next, we analyzed rostral brain DA levels and the number of DA neurons in the substantia nigra pars compacta. We found that at P7.5, DA levels in the rostral brain were increased by 25% to a similar extent in both *Gdnf* ^*wt/hyper*^ and *Gdnf* ^*hyper/hyper*^ mice, and normalizing *Gdnf* levels by crossing the mice with Deleter-FLP animals restored DA to wild-type levels (Fig 4B). Compared to wt mice, the number of DA neurons in the substantia nigra pars compacta revealed a similar 15% increase in *Gdnf* ^*wt/hyper*^ and *Gdnf* ^*hyper/hyper*^ animals at P7.5 (Fig 4C). Finally, the levels of DA metabolites in the rostral brain were similar between genotypes at P7.5 (S3A and S3B Fig). The development of the DA system was not linked with kidney development, as kidney function was severely impaired in the *Gdnf* ^*hyper/hyper*^ mice, but not in the *Gdnf* ^*wt/hyper*^ mice while the DA system parameters were comparable between the *Gdnf* ^*wt/hyper*^ and *Gdnf* ^*hyper/hyper*^ mice. This finding was further supported by the lack of correlation between serum urea and brain DA levels in individual heterozygous animals at P7.5 (S3 Table). We conclude that increased GDNF levels increase striatal DA levels and DA cell number in the substantia nigra pars compacta at P7.5.

### Increased GDNF levels increase the nigrostriatal dopaminergic system in adult mice

To assess whether DA system was changed in adult animals, we studied Gdnf^wt/hyper^ mice at 2.5–4 months of age and noted that the DA levels in the striatum of *Gdnf* ^*wt/hyper*^ mice were increased by 25% compared to wild-type littermates (Fig 4D). Analysis of striatal DA metabolites (S3C and S3D Fig) revealed a 35–40% increase in 3,4-dihydroxyphenylacetic acid (DOPAC) levels (S3C Fig), suggesting that also DA release in *Gdnf* ^*wt/hyper*^ mice may be increased. To assess the effect of the absence of one *Gdnf* allele in the comparable genetic background, we also measured DA levels in heterozygous GDNF-knockout mice (*Gdnf* ^*wt/KO*^) [10]. Consistent with previous studies [21] the levels of DA in *Gdnf* ^*wt/KO*^ mice were similar to wild-type levels (Fig 4D). Restoring wild-type expression levels of GDNF by crossing *Gdnf* ^*wt/hyper*^ mice with Deleter-FLP mice also restored DA levels (S3E Fig). In the substantia nigra pars compacta the number of DA neurons in the *Gdnf* ^*wt/hyper*^ mice was 15% increased relative to the controls (Fig 4E and 4F), indicating that the moderate 15% increase in the number of substantia nigra pars compacta DA neurons noted at P7.5 is retained in adulthood. Similarly, we found that the number of dopaminergic terminals in the dorsal striatum is increased by 15% in the *Gdnf* ^*wt/hyper*^ mice (Fig 4G, S3F Fig). Immunohistochemical examination of nigrostriatal DA system revealed no gross anatomical differences between the *Gdnf* ^*wt/hyper*^ and *Gdnf* ^*wt/wt*^ mice (S3G and S3H Fig) and the size of the striatum appeared unaffected (S3I Fig).

Collectively, these data indicate that an increase in GDNF expression levels increases the adult number of DA cells in the substantia nigra pars compacta and the number of dorsal striatal DA terminals by 15%. In addition, increase in GDNF expression increases striatal tissue DA and its metabolite DOPAC levels by 25% and 35–40%, respectively, suggesting that DA release in *Gdnf* ^*wt/hyper*^ mice may be enhanced.

### Increased GDNF levels increase specific DA system functions in adult mice

To determine whether increasing the levels of endogenous GDNF affects the function of the nigrostriatal DA system in adult mice, we performed fast-scan cyclic voltammetry measurements in acute striatal slices at 3–4 months of age, and measured the clearance rate of extracellular exogenous DA in the striatum using *in vivo* amperometry at the same age. To study various functional aspects of the DA system, we used a range of stimulation patterns for the voltammetry measurements (S3J Fig). We observed that stimulations in the striatal tissue of *Gdnf* ^*wt/hyper*^ mice released about 35–45% more DA (Fig 4H and 4I) with a steeper rising slope (S3K Fig) when compared to *Gdnf* ^*wt/wt*^ mice. No differences were found between the genotypes in the rise time and decay parameters of the DA events (S3L–S3N Fig). We observed no difference in paired stimulus depression of DA release (Fig 4J), and we found no difference in DA release probability (Fig 4K).

Since more DA is released but DA reuptake parameters are comparable between the genotypes, increased DA uptake in the striatum of *Gdnf* ^*wt/hyper*^ mice can result at least in part, from the observed 15% increase in the number of dopaminergic terminals in the striatum. However, increased DA transporter (DAT) levels and/or increased DAT activity could also contribute to the observed increase in DA re-uptake. To test these possibilities, we measured the clearance rate of extracellularly applied DA in the striatum using *in vivo* amperometry. We found that compared to *Gdnf* ^*wt/wt*^ mice, DAT activity in the *Gdnf* ^*wt/hyper*^ mice was increased at least up to five-fold in a DA concentration-dependent manner (Fig 4L). Next, we examined whether this difference is due to differences in the levels of DAT between genotypes. We measured total and surface levels of DAT protein in the striata at P7.5 and total DAT levels in the striata of adult mice; we found no differences between the genotypes (S4A–S4C Fig). Taken together, our findings suggest that increasing endogenous GDNF levels increases striatal tissue DA content and striatal DA release and re-uptake without affecting the DA release probability. The observed increase in striatal DA re-uptake in *Gdnf* ^*wt/hyper*^ mice is most likely explained by the combined effects of increased DAT activity and 15% increase in the number of striatal dopaminergic terminals.

### *Gdnf* ^*wt/hyper*^ mice have an increased response to amphetamine

In order to investigate whether increased GDNF levels result in functional consequences in the DA system in behaving animals we administered amphetamine, a dopaminergic stimulant. Amphetamine is taken up by the DAT into the presynaptic terminal, where it is then loaded into synaptic vesicles. Amphetamine releases DA from these vesicles into the cytoplasm and reverses its active transport across the presynaptic membrane; the result is increased DA concentration in the synaptic cleft and increased locomotor activity in treated animals [22]. Compared to *Gdnf* ^*wt/wt*^ littermates, *Gdnf* ^*wt/hyper*^ mice had increased amphetamine-induced locomotor activity (Fig 4M), suggesting that amphetamine has a stronger effect in terms of driving higher extracellular DA levels in the striatum of *Gdnf* ^*wt/hyper*^ mice. To test this hypothesis, we measured extracellular dopamine levels in the striatum following local amphetamine delivery via *in vivo* microdialysis. Consistent with our hypothesis, we observed an increase in amphetamine-induced extracellular DA levels in the striata of *Gdnf* ^*wt/hyper*^ mice (Fig 4N). We also measured the effect of amphetamine in striatal slices using cyclic voltammetry. Because amphetamine depletes DA from nerve terminals, cyclic voltammetry can detect the gradual decrease in stimulated DA release following amphetamine application. Consistent with increased DAT function in *Gdnf* ^*wt/hyper*^ mice, we found that amphetamine depletes the synaptic DA storages with a faster time course in *Gdnf* ^*wt/hyper*^ mice compared to *Gdnf* ^*wt/wt*^ littermate controls (Fig 4O).

Based on these results, the increased amphetamine-induced locomotor activity in *Gdnf* ^*wt/hyper*^ mice is likely due to a combination of three effects. First, the increased DAT activity in *Gdnf* ^*wt/hyper*^ mice likely causes amphetamine to accumulate in the nerve terminals more rapidly. Second, the 15% increase in the number of striatal dopaminergic terminals increases the number of striatal DA release sites. And third, more DA is released by the terminals. Collectively, these DA system features explain the increased amphetamine-induced locomotor activity in *Gdnf* ^*wt/hyper*^ mice.

### Increasing GDNF levels protects the dopaminergic system in a lactacystin-induced model of Parkinson’s disease

Currently, no mouse model is available that phenocopies the slow disease progression of patients with PD [23]. Thus, the most widely used animal models for studying PD are based on the toxins MPTP (1-methyl-4-phenyl-1,2,3,6-tetrahydropyridine) and 6-hydroxydopamine (6-OHDA), both of which are taken up specifically into DA neurons via the DAT [23]. Given that we found at least up to five-fold increase in DAT activity in *Gdnf* ^*wt/hyper*^ mice, we expect *Gdnf* ^*wt/hyper*^ mice to be sensitized to DAT-based toxins, unless the trophic effect of increased endogenous GDNF expression dampens or reverses the phenotype. We found that relative to the controls, *Gdnf* ^*wt/hyper*^ mice are five-fold more sensitive to striatal 6-OHDA injection. More specifically, following striatal 6-OHDA injection we found five-fold aggravated decrease in striatal DA levels and two-fold aggravated decrease in substantia nigra pars compacta DA neuron numbers in *Gdnf* ^*wt/hyper*^ mice relative to the littermate controls (Fig 4P and 4Q). To overcome the confounding effect of enhanced DAT activity in *Gdnf* ^*wt/hyper*^ mice in DAT based PD models, we looked for alternative models of PD. Abnormal aggregation of proteins is a generally accepted pathological process common to most neurodegenerative disorders, including PD. Consistent with this notion, intracranial application of proteasome inhibitors such as lactacystin can induce a PD-like phenotype in both rodents and fish [24,25]. We found that unilateral lactacystin injection just above the substantia nigra induced significant side bias (measured using the corridor test) in *Gdnf* ^*wt/wt*^ mice; however, lactacystin-injected *Gdnf* ^*wt/hyper*^ mice did not develop this bias (Fig 4R). In addition, DA and its metabolite levels were better preserved in the striatum of lactacystin-injected *Gdnf* ^*wt/hyper*^ mice compared to lactacystin-injected wild-type mice (Fig 4S). However, the number of DA cells in the substantia nigra pars compacta was comparably reduced by lactacystin injection in both genotypes (Fig 4T), suggesting that the protective effect from lactacystin-induced PD in *Gdnf* ^*wt/hyper*^ mice occurs at the functional level in the striatum.

### GDNF derepression does not induce side effects associated with ectopic GDNF application

Despite its dopaminotrophic benefits both in PD models and in clinical trials, the delivery of GDNF to the nigrostriatal DA system also induces adverse side effects, including hyperactivity [5,26–29], reduced levels of striatal tyrosine hydroxylase (TH)–the rate-limiting enzyme in DA synthesis [5,30], reduced food intake, and loss of body weight [6,26]. Importantly, none of these effects were observed in our *Gdnf* ^*wt/hyper*^ mice as assessed by open field test, measurements of food intake in physiological cage, bodyweight measurements of adult mice at 3–4 months of age (Fig 4U–4W) and by western blotting and immunohistochemical measurements of striatal TH levels at P7.5 and in adult mice at 3–4 months of age (S4D–S4G Fig).

### miR-9, miR-96, miR-133 and miR-146a are novel regulators of GDNF

**Fig 5:**
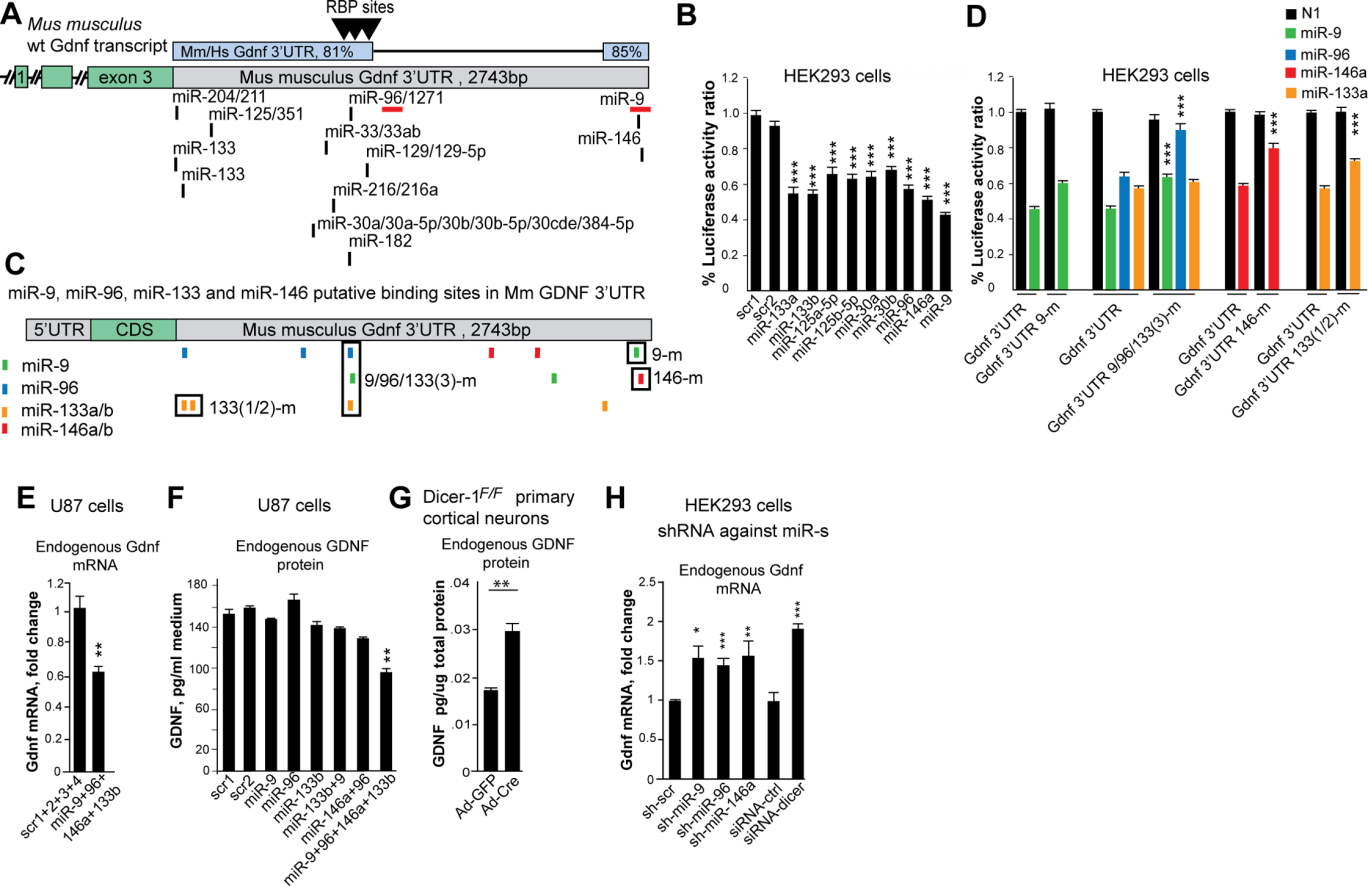
Identification of *Gdnf*-regulating miRNAs. **(A)** Putative miR binding sites cluster within the conserved areas of the *Gdnf* 3’UTR. The miRNAs underlined with red bars were co-immunoprecipitated with *Gdnf* mRNA in a genome-wide screen of a mouse brain tissue [34]. Predicted putative RNA-binding protein (RBP) sites are indicated with triangles. Source: Blast, TragetScan, AREsite, and http://servers.binf.ku.dk/antar/; Hs, human; Mm, mouse. **(B)** Luciferase expression from a Ren-*Gdnf* 3’UTR construct after co-transfection with the indicated pre-miRNAs in HEK293 cells. scr1 and scr2 are scrambled pre-miRNA controls; N = 3 experiments/miRNA with 3–5 biological repeats/miRNA/experiment. **(C)** Predicted binding sites (ddG<-3) for miR-9 (green), miR-96 (blue), miR-133a/b (orange), and miR-146a (red) based on “predict microRNA targets” analysis (http://genie.weizmann.ac.il/). Boxes indicate the mutated miRNA-binding sites in each mutant. Note that miR-9/96/133m contains overlapping sites for miR-9, miR-96, and miR-133, all of which were mutated in this construct. **(D)** Luciferase assay of miR-9, miR-96, miR-133a, and miR-146a mutants (mutated sequences indicated with boxes in panel C; scr1 is a scrambled pre-miRNA control; N = 2 experiments/miRNA with 3 biological repeats/miRNA/experiment. **(E-F)** Expression of endogenous *GDNF* mRNA (E) and GDNF protein (F) is inhibited in U87 cells by co-transfection with the indicated pre-miRNAs; N = 3–4 experiments with 2–3 biological replicates/experiment. **(G)** Adenoviral transduction of Cre—but not GFP—in homozygous “floxed” Dicer-1^F/F^ primary cortical neurons increases endogenous GDNF protein levels; N = 2 experiments with 3–4 mice/group. **(H)** AAV-based constructs encoding shRNAs against miR-9, miR-96, and miR-146a, as well as siRNA against Dicer, increase endogenous *GDNF* expression in HEK293 cells; N = 5 experiments/construct (except for the shRNA against miR-9, where N = 2 experiments/construct) with 2 biological repeats per experiment. Ad, adenovirus; m, mutant.

To get insight on the mechanism how *Gdnf* 3’UTR negatively regulates gene expression (S1D–S1F Fig) we looked for *trans*-acting factors that regulate transcripts containing the *Gdnf* 3’UTR. Sequence analysis revealed three putative binding sites for RNA-binding proteins (RBPs) and conserved binding sites for several miRNAs in the *Gdnf* 3’UTR (Fig 5A). We found that the RBPs tristetraprolin (TTP, a negative regulator of brain-derived neurotrophic factor BDNF [31]); embryonic lethal abnormal vision-like protein 1 (ELAVL1); and the AU-rich element-binding protein AUF1 had little or no effect on the expression of a reporter construct containing the *Gdnf* 3’UTR (S4H Fig), consistent with a previous report [11].

Next, we investigated the effect of miRNAs on regulating *Gdnf* expression. We examined the miRNAs miR-133a, miR-133b, miR-125a-5p, miR-125b-5p, miR-30a, miR-30b, miR-96, miR-9, and miR-146a, which were selected based on their co-expression with *Gdnf* in several brain areas [17,19,32,33]; see also www.microrna.org and presence of conserved binding sites within *Gdnf* 3’UTR (Fig 5A; www.targetscan.org). Our analysis of miRNA expression revealed that miR-9, miR-133a, miR-133b, miR-125a-5p, miR-125b-5p, miR-30a, miR-30b, and miR-146a are all expressed in the developing forebrain, adult dorsal striatum and in the developing kidney (S4 Table). Next, we transfected HEK293 cells with the above-mentioned putative *Gdnf*-regulating pre-miRNAs. Compared to the control miRNAs, the specific miRNAs negatively regulated the expression of a reporter construct containing the *Gdnf* 3’UTR by 30–50% (Fig 5B). Next, we examined whether the *in silico* (genie.weizmann.ac.il, www.targetscan.org) predicted conserved miRNA sites mediate the interaction between these miRNAs and the *Gdnf* 3’UTR. We paid particular interest to miR-9 and miR-96, as previous data obtained from two genome-wide screens suggested that these miRNAs interact with *Gdnf* mRNA in the mouse brain; and overexpressing them in human cell line suppresses the expression of *GDNF* (summarized in [34]). Mutating some of the predicted miRNA seed sites (Fig 5C; see S1 Materials and Methods for details) in the *Gdnf* 3’UTR either reduced or abolished the ability of miR-9, miR-96, miR-133a, and miR-146a to inhibit expression (Fig 5D), suggesting a direct interaction between these miRNAs and some of the predicted sites in the *Gdnf* 3’UTR. Based on *in silico* analysis, puΔtk contains approximately half the number of potential miRNA-binding sites and about 10% of the conserved miRNA sites present in the *Gdnf* 3’UTR (S4I and S4J Fig). Consistent with this prediction, we found that miRNAs had no effect on expression of a reporter construct containing the *puΔtk-Gdnf 3’UTR* cassette (S4K Fig). To analyze the effect of miR-9, miR-96, miR-133b, and miR-146a on endogenous *GDNF* expression, we transiently overexpressed these miRNAs in U87 cells (a human glioblastoma cell line that expresses endogenous GDNF at detectable levels). We found that compared to the control miRNAs, transient co-expression of these four miRNAs reduced the *Gdnf* mRNA level (Fig 5E) and GDNF protein level (Fig 5F) without affecting cell survival (S4L Fig).

Dicer is required for the maturation of miRNAs. To gain further evidence that the GDNF expression is regulated by miRNAs, we next examined the effect of deleting Dicer on endogenous GDNF expression in primary Dicer^flox/flox^ cortical neurons [35] using adenovirus-mediated delivery of Cre. Deleting Dicer resulted in up-regulation of endogenous GDNF expression in cortical neurons (Fig 5G). Finally, to gain insight into how endogenous miR-9, miR-96 and miR-146a impact endogenous *GDNF* mRNA levels in human cells we tested six different shRNA constructs targeted to each miRNA for their ability to derepress endogenous GDNF expression in HEK293 cells (S4M Fig). Based on this analysis, we identified the three shRNA constructs targeting miR-9, miR-96 and miR-146a with the highest potency for derepressing endogenous *GDNF* mRNA expression (Fig 5H). An siRNA against Dicer was used as a positive control (Fig 5H). We conclude that miR-9, miR-96, miR-133, and miR-146a interact directly with their binding sites in the *Gdnf* 3’UTR; moreover, miR-9, miR-96, and miR-146a regulate the expression of GDNF *in vitro*.

## Discussion

Due to the limitations associated with existing genetic tools, the function of endogenous GDNF has remained poorly understood [7,10]. Despite multiple attempts by several research groups, transgenic animals in which GDNF expression is restricted to cells that normally express GDNF are not available. The dramatic consequences of ectopic GDNF expression—for example, on the development of the urogenital tract [36]—make it difficult to draw conclusions regarding the function of endogenous GDNF based solely on ectopic GDNF expression in the brain.

### Analysis of GDNF hypermorphic mice

GDNF hypermorphic mice provided us with an opportunity to study the function of endogenous GDNF with the focus on the postnatal nigrostriatal DA system and in renal development.

We found that increasing the endogenous levels of GDNF increases the number of DA neurons in the substantia nigra pars compacta during the development, and that this increase in DA neuron numbers is retained in adulthood. Highlighting the importance of the correct expression site, GDNF has no such effects when overexpressed in the mouse brain under the promoter not specific to GDNF-expressing neurons [8].

In the dorsal striatum, increase in GDNF levels resulted in increased number of dopaminergic terminals, increased levels of DA and enhanced DA release and reuptake, explaining the enhanced amphetamine-induced locomotor activity in GDNF hypermorphic mice. Since the nigrostriatal DA system is well known for its capacity to compensate for changes [37], the observed increase in DA transporter activity in GDNF hypermorphic mice likely reflects a measure to counterbalance elevated striatal DA content and release to maintain normal extracellular DA levels. We observed no change in striatal DA transporter levels, suggesting that DAT activity in GDNF hypermorphic mice is regulated by other mechanisms, such as post-translational modifications or protein-protein interactions. Since currently methods allowing temporal analysis of those endpoints in the mouse striatum are not available, the question of how GDNF regulates DAT activity remains to be resolved.

Notably, we found that increased GDNF levels have a protective effect in supranigrally delivered lactacystin model of Parkinson’s disease; this effect is not mediated by protecting DA cell bodies in the substantia nigra pars compacta, but by enhancing the dopaminergic function in the striatum. These findings are consistent with a previous study reporting the lack of effect of selective GDNF deletion or reduction on the survival of DA cell bodies in the substantia nigra pars compacta upon aging [10] and with a study reporting that deletion of GDNF receptor RET does not modulate MPTP toxicity on the dopaminergic system but is required for regeneration of striatal dopaminergic axon terminals [38]. Collectively, these results suggest that in adult mice GDNF acts as a local trophic factor for DA axons in the striatum.

Furthermore, our *Gdnf* ^*wt/hyper*^ mice do not develop any of the adverse side effects usually associated with ectopic GDNF expression, which can include hyperactivity, loss of body weight, and a decrease in the levels of tyrosine hydroxylase—the rate-limiting enzyme in DA synthesis. Because GDNF is not applied ectopically in our model, the common feature in both experimental animals and human studies—specifically, the massive sprouting of DA fibers towards the site of GDNF delivery, with unknown consequences with respect to side effects, treatment efficacy and behavior—was not observed in *Gdnf* ^*wt/hyper*^ mice. Together, these results imply that measures that promote elevation in endogenous GDNF levels in the striatum may have clinical potential in the treatment of Parkinson’s disease. However, because we found increased DAT activity in GDNF hypermorphic mice, the simultaneous use of GDNF with DAT-based toxins or drugs should be carefully considered and calls for further investigation.

To date, data on GDNF function is mainly gathered using various ectopic GDNF application methods in rodent and primate models and using constitutive or conditional GDNF knock-out mice. Our results bring an important new dimension since we analyze the effect of the elevation of endogenous GDNF. In Table 1 we illustrate the qualitative difference between ectopic and endogenous GDNF sources.

**Table 1:** Summary of GDNF functions identified using GDNF hypermorphic mice. The main findings of this study are reported in comparison with published reports from ectopic GDNF applications and GDNF knock-out mice. The forth column indicates whether the observations made in GDNF hypermorphic mice are consistent with function predicted from ectopic GDNF applications and from *Gdnf* gene deletion studies, expand the previous knowledge or provide novel information.

Endogenous GDNF elevation (this study)	Reported ectopic GDNF expression or gene knock-out approaches		Novelty of the finding
Observed phenotype	Observed phenotype	Approach	
Increased motor activity upon amphetamine injection	Increased motor activity upon amphetamine injection	Nigrostriatal recombinant GDNF injection and viral gene delivery [5,26]	Consistent with ectopic/KO approaches
GDNF controls DA cell number at P7.5	GDNF controls DA cell number at P7.5	2–3 fold transgenic over-expression of GDNF in the forebrain using CaMKIIα promoter [8]	Consistent with ectopic/KO approaches
Striatal DA levels are maintained upon supra-nigral lactacystin injection	Striatal DA levels are maintained upon supra-nigral lactacystin injection	Striatal viral gene delivery [39]	Consistent with ectopic/KO approaches
Increased stimulated and amphetamine -induced DA release	Increased stimulated and amphetamine -induced DA release	Intranigral GDNF protein injection [27,28]	Consistent with ectopic/KO approaches
GDNF levels regulate kidney size and morphological maturation	GDNF is required for the induction of the ureteric bud development during early embryogenesis	GDNF gene deletion [15]	Consistent and expanding ectopic/KO approaches
Elevated GDNF increases the number of DA neurons and enhances DA system functionduring development and in adulthood	GDNF deletion or reduction has no effect on the number of DA neurons in adulthood and upon aging; no gross effect on animal behavior	Conditional GDNF deletion or reduction using three Cre lines [10]	Novel
Elevated GDNF increases the number of DA neurons during development which is retained in adulthood.	Elevated GDNF does not increase the number of DA neurons after two weeks of age	2–3 fold transgenic over-expression of GDNF in the forebrain using CaMKIIα promoter [8]	Novel
Elevated GDNF increases striatal DA release and re-uptake	Elevated GDNF has no effect on DA release and re-uptake	2–3 fold transgenic over-expression of GDNF in the forebrain using CaMKIIα promoter [8]	Novel
No effect on food intake and bodyweight at least until 3–4 months of age	Reduction in food intake and weight loss within 1–2 weeks after GDNF overexpression	Nigrostriatal recombinant GDNF injection and viral gene delivery [6,26]	Novel
No effect on striatal tyrosine hydroxylase levels at least until 3 months of age	Downregulation of striatal tyrosine hydroxylase levels after 6 weeks	Nigrostriatal viral gene delivery [5,30]	Novel
No effect on spontaneous motor activity	Spontaneous motor hyperactivity	Nigrostriatal recombinant GDNF injection [26–29]	Novel
Elevation in total striatal DA levels	; No effect on total striatal DA levels; elevation in DA turnover	Nigrostriatal recombinant GDNF injection and viral gene delivery [5,26,28]	Novel
No aberrant sprouting of DA fibers	Sprouting of DA fibers towards GDNF injection site	Nigrostriatal recombinant GDNF injection and viral gene delivery [4,26,40]	Novel
Elevated GDNF augments DA concentration -dependent increase in DAT activity	Not addressed previously		Novel

With respect to the role of GDNF in renal development, we found that excess GDNF levels negatively regulate kidney growth and morphogenesis. This result is contrary to expectations based on the majority of *in vitro* studies, where the effect of ectopic GDNF on embryonic urogenital block can be followed for the few days, and likely arose from an overstimulation of ureteric bud growth, a key process in renal development.

A comparison between our *Gdnf* ^*wt/hyper*^ mice and MEN2B mice, which express a constitutively active form of the GDNF receptor RET [41], revealed several common features and key differences between these two models. For example, striatal DA levels, TH-positive cell numbers, and striatal DAT activity are increased in both mutants. In contrast, only MEN2B mice develop increased levels of DAT and TH, and reduced spontaneous locomotion [42–44]. Thus, increasing endogenous GDNF levels is fundamentally different than constitutively activating RET; this finding may have broad-reaching implications with respect to drug design and studies of receptor-ligand biology.

### miRNAs and regulation of GDNF expression via the 3’UTR

Previously published genome-wide screens suggested that overexpressing miR-9 and miR-96 reduce the levels of *GDNF* mRNA in a human cell line and that these miRNAs interact with the *Gdnf* mRNA in the mouse brain [34]. We identified binding sites for miR-9 and miR-96 in the 3’UTR of *Gdnf*; in addition, we identified binding sites for miR-133 and miR-146a. We also found that reducing the levels of Dicer, an enzyme required for the maturation of miRNAs, derepresses endogenous GDNF expression both in human cells and in mouse primary neurons. Moreover, overexpressing miR-9, miR-96, miR-133b, and miR-146a represses the expression of endogenous GDNF mRNA and protein in a human cell line. Finally, we found that shRNAs against miR-9, miR-96, and miR-146a derepress endogenous *GDNF* mRNA levels in human cell line. Taken together, these results confirm the miRNA target *in vitro* [45]. However, every miRNA can have several hundred mRNA targets; thus, assigning specific observations to the direct effect of a given miRNA acting on one target *in vivo* is currently not possible. Future work with a conditional targeting of *Gdnf* 3’UTR is necessary to overcome this limitation. Once the effects of adult-onset GDNF elevation in the striatum are characterized, we can then utilize various anti-miRNA strategies to evaluate a given miRNA’s role in GDNF-induced changes in the DA system. Our data also suggest that miR-125a-5p, miR-125b-5p, miR-30a, and miR-30b are possible regulators of GDNF expression. Whether the effect of these miRNAs on GDNF expression is direct or indirect warrants further research.

Interestingly, we found that GDNF derepression in GDNF hypermorphic mice was stronger in the kidneys than in the brain and in the brain areas in which *Gdnf* mRNA levels are higher. The reasons underlying this finding are currently unknown; however, it may be related to the relative ratio between the mRNA and miRNAs; in addition, miRNAs can have different effects on their target mRNAs depending on the tissue context [46].

### Conclusions

Using GDNF hypermorphic mice we found that endogenous GDNF regulates postnatal development and function of nigrostriatal dopamine system. Moreover, some of the identified GDNF functions overlapped with results from earlier studies with ectopic GDNF, whereas others were novel, highlighting the importance of correct spatial expression of GDNF. We also found that about two-fold elevation in endogenous GDNF levels protects mice in lactacystin-based model of Parkinson’s disease without side effects associated with ectopic GDNF applications. Whether increasing endogenous GDNF levels is a viable strategy for developing new therapeutic approaches for treating Parkinson’s disease and other diseases is an important question. Finally, since negative regulation via 3’UTR is shared by many genes, our data pinpoints that 3’UTR-s could provide an important target for genetic studies *in vivo*. More specifically, prevention of transcription of negatively regulated 3’UTR-s could provide a measure to elevate endogenous gene expression while avoiding mis-expression commonly associated with transgenesis.

## Materials and Methods

Detailed descriptions of all materials and methods are provided in S1 Materials and Methods.

### Ethics statement

The animal experiments were performed according to the EU legislation harmonized with Finnish legislation and have been approved by the National Animal Experiment Board of Finland (permit no. ESAVI/3770/04.10.03/2012).

### Cell culture and molecular biology assays

Cell culture and molecular biology assays were performed using routine methods in the field; please see S1 Materials and Methods for details.

### Generation of GDNF hypermorphic animals, tissue dissection and analysis

Briefly, 5667bp 5’ homologous arm spanning the second intron of the *Gdnf* gene, 6055bp 3’ homologous arm and GDNF protein coding part of *Gdnf* exon 3 including the stop codon were amplified with PCR from *Gdnf*-containing PAC (RP21-583-K20, CHORI) and cloned into pFlexible [12,47], to generate *Gdnf* targeted allele**.** ES clones were screened with standard Southern blotting. Mice were maintained in 129Ola/ICR/C57bl6 mixed genetic background.

### Lactacystin model of Parkinson’s disease

4 μg of lactacystin (AG Scientific) in 4 μl of PBS was injected just above the SN at: antero-posterior (AP) -3.3 mm; medio-lateral (ML) -1.2 mm and dorso-ventral (DV) -4.6 mm. The animals were subjected to corridor test 5 weeks after the injection, and sacrificed for tissue isolation and IHC analysis.

### *In situ* hybridization

*In situ* hybridization was performed using a probe spanning *Gdnf* exons or 525bp in the 3’ end of *Gdnf* 3’UTR. RNAscope [20] probes detecting *Gdnf* (red) and *Parvalbumin (PV*, blue) mRNA were custom made by Advanced Cell Diagnostics.

### Fast-scan cyclic voltammetry

Dopamine release was evoked on acute striatal slices with electrical stimulations and measured with a carbon fiber electrode calibrated with known dopamine concentrations. The signal was amplified with Axopatch 200B amplifier (Molecular Devices), digitized (ITC-18 board; InstruTech) and analyzed with a computer routine in IGOR Pro (WaveMetrics).

### *In vivo* chronoamperometry

In anesthetized mice (urethane 1.7–1.9 g/kg, i.p.; Sigma) the electrode was mounted in parallel with a micropipette used for application of dopamine. Recordings were performed at two rostrocaudal striatal tracks in each hemisphere, at AP +0.3 or +1.0 mm; ML ±1.8 mm, using Fast Analytical Sensing Technology (FAST-16) system (Quanteon). At each recording site, data was collected from three depths below the dura: at -2.0, -2.5, and -3.0 mm.

### *In vivo* microdialysis

A microdialysis guide cannula (MAB 4.1, AgnTho’s AB) was inserted into the dorsal striatum (AP +0.6 mm; ML +1.8 mm and DV -2.2 mm) of mice under isoflurane anaesthesia. After obtaining a stable baseline, the Ringer solution was switched into 100 μM of D-amphetamine for 60 minutes. The dialysis flow rate was 2 μl/min. Concentration of dopamine was analyzed using HPLC.

### Measurements of GDNF protein levels from tissues

Striatal tissues lysates were prepared and analyzed immediately after sacrificing with GDNF Emax ImmunoAssay System (Promega).

### Behavioral analyses

Open field test was performed in three independent cohorts of comparable size (N = 10–12 male mice per genotype with littermate controls). Metabolic monitoring was performed using Comprehensive Lab Animal Monitoring System (CLAMS).

### Statistical analysis

Statistical analysis for pairwise comparisons was performed using Student’s t-test with two tailed distribution using the unequal variance option. Data from amperometry was analyzed by one-way ANOVA followed by Bonferroni *post hoc* test. Behavioral data were analyzed using factorial ANOVA design with genotype and cohort as between-subject factors, where appropriate. *Post hoc* analysis after significant ANOVA was carried out using Student-Newman-Keuls test. Data from CV was analyzed by two-way repeated measures ANOVA, which in the amphetamine analysis was followed by multiple comparisons (Sidak´s). All numerical results are reported as mean ± standard error of the mean. SPSS (IBM Corp., Armonk NY, USA) or STATISTICA 11 (StatSoft Inc., Tulsa) were used for analysis.

## Supporting Information

S1 Materials and Methods Detailed description of materials and methods used in this study. (DOCX)

S1 Table GDNF levels regulate kidney size and number. Gdnf KO animals were obtained by crossing *Gdnf* ^*wt/hyper*^ animals to Deleter-Cre mice, which results in deletion of GDNF protein coding exon 3 (Fig 1B) [10].
(DOCX)

S2 Table *Gdnf* ^*hyper/hyper*^ mice die before weaning, whereas *Gdnf* ^*wt/hyper*^ mice are produced in Mendelian ratios. (DOCX)

S3 Table Lack of correlation between serum urea and rostral brain dopamine levels in individual *Gdnf* ^*wt/hyper*^ mice. Correlational analyses of rostral brain dopamine and serum urea levels in individual animals in 10 *Gdnf* ^*wt/wt*^ and 12 *Gdnf* ^*wt/hyper*^ animals at P7.5 using the Correlation function in Microsoft Excel.
(DOCX)

S4 Table miR expression levels in different tissues and cell lines. Levels of mature miRs expressed as fold difference relative to sno202 in adult mouse brain, developing mouse kidney and in HEK293 cells. N = 2 experiments. dSTR, dorsal striatum, E, embryonic day; Hs, *Homo sapiens*; Mm, *Mus musculus*; P, postnatal day; w, weeks.
(DOCX)

S1 Fig *In vitro* analysis of *Gdnf* 3’UTR. **(A)** Schematic representation of the reporter constructs and the derived mRNAs used in this study. Red and blue bar indicate probes used in the Northern blot for firefly luciferase CDS and *Gdnf* 3’UTR, respectively. **(B)** The *puΔtk* cassette blocks transcription to the *Gdnf* 3’UTR, measured by Northern blot analysis of expression from a construct harboring the Firefly (FF) coding sequence upstream of the *puΔtk-Gdnf* 3’UTR cassette in HEK293 cells; loading control: 28S ribosomal RNA. **(C)** Northern blot analysis of expression from construct harboring FF coding sequence proceeded with *puΔtk-Gdnf* 3’UTR cassette (S1 Fig A) in HEK293 cells (S1 Fig D); loading control: 28S ribosomal RNA. **(D)** Expression from equimolar amounts of the FF-*Gdnf* 3’UTR, FF-*puΔtk-Gdnf* 3’UTR and FF-SV40 pA constructs in U87 and HEK293 cells; FF-firefly luciferase; N = 3 experiments/construct with 3 replicates/experiment. **(E)** *Gdnf* 3’UTR reduces expression of both Renilla luciferase (Ren) and FF in HEK293 and U87 cells; N = 3 experiments/construct with 3 replicates/experiment **(F)** FF expression from constructs containing SV40 pA, *puΔtk-Gdnf* 3’UTR cassette and *Gdnf* 3’UTR in HEK293 cells after treatment with actinomycin D. Note that prevention of transcription of the native *Gdnf* 3’UTR by preceding *puΔtk* alleviates the post-transcriptional inhibition of the reporter. *Renilla* luciferase expression from a separate plasmid was used for normalization. N = 3 experiments/construct with 3 replicates/experiment. **(G)** Targeting strategy of the *Gdnf* locus. 5667 bp 5’ (green bar) and 6055 bp 3’ (blue bar) homologous arms, and *Gdnf* exon 3 until and including the stop codon were amplified with PCR from *Gdnf*-containing PAC and cloned into *Pme*I, *Not*I and *Hind*III sites in pFlexible [31], respectively, to generate *Gdnf* targeted allele. Sequence lengths are not drawn to scale**.** Arrows indicate primers used for routine genotyping; black bar indicates probe used for Southern blotting using BlpI restriction enzyme shown on **(H)**. **(I)** Representative image of routine genotyping of the mice using primers indicated on G. Abbreviations: *puΔtk*, a cassette with PGK promoter encoding for fusion protein between puromycin N-acetyltransferase and a truncated version of herpes simplex virus type 1 thymidine kinase (TK) with 3’ bovine growth hormone polyadenylation signal (pA); FF-firefly luciferase; CDS-coding sequence.
(TIF)

S2 Fig Analysis of *Gdnf* expression and kidney function in *Gdnf* hypermorphic mice. **(A-B)** Representative images of *in situ* hybridization of *Gdnf* mRNA using a probe against the CDS (stained blue, indicated with white arrow heads) in mice in whole-mount preparations of E11.5 hindlimb (A) and in paraffin sections from P7.5 testis (B). Note that *Gdnf* expression sites are comparable between genotypes, whereas the signal appears stronger in *Gdnf* ^*hyper/hyper*^ mice. N = 4 mice/group in A and N = 2 in B. **(C-D)** *Gdnf* mRNA (C) and protein (D) expression in the testis at E18.5 measured with QPCR and ELISA, respectively, shows allele dose-dependent increase in GDNF levels in GDNF hypermorphic mice. Note that GDNF protein levels in *Gdnf 3’UTR* ^*rest/rest*^ mice are normal (D). N = 2–5 mice/group in 2–3 experiments with 2–3 replicates/experiment **(E)** Representative image of Northern blot analysis of *Gdnf* mRNA in E18.5 testis using *Gdnf* CDS probe. Note that the size of the band is ca 500 bp shorter in *Gdnf* ^*hyper/hyper*^ mice compared to the *Gdnf* ^*wt/wt*^ mice, consistent with the size of transcripts derived from *Gdnf-puΔtk* fusion transcript (S1A–S1C Fig; and see S2 Fig F, G). N = 2 mice/group. **(F)** Schematic representation of the *puΔtk-Gdnf 3’UTR* (*Gdnf* ^*hyper*^) allele for *Gdnf*, *A* and *B* designate primers used in QPCR analysis of *Gdnf* mRNA levels (C); primers *A* and *C* depict primers used for *Gdnf* transcript sequence analysis in (G). **(G)** Representative image of RT-PCR analysis from E18.5 testis using primers *A* and *C* as depicted in (F). The observed PCR product in *Gdnf* ^*hyper/hyper*^ mice was of expected length (977 bp) and it was sequenced for validation; N = 3 mice; beta-actin was used to verify cDNA quality. **(H-I)** Kidneys of P7.5 *Gdnf* ^*hyper/hyper*^ mice function poorly, as indicated by serum creatinine (H) and serum urea (I) levels. H, N = 3–12; I, N = 7–26 mice/group. **(J-K)** The blood serum of adult *Gdnf* ^*wt/hyper*^ mice contains slightly higher levels of urea (J), but not creatinine (K) compared to wild type mice. Note that two-fold variation in serum urea levels is considered normal in humans. N = 16–19 mice/group. **(L)** Representative image of kidneys in wild type and *Gdnf* 3’UTR^rest/rest^ mice. In total, 20 mice were analyzed from each genotype. **(M)** Verification of the specificity of RNAscope probes against *parvalbumin* (left panel; blue) and *Gdnf* (right panel; red). Note that the signal matches known *parvalbumin* mRNA expression in Purkinje cells (black triangles) and in molecular layer interneurons (open triangles) at three months of age. Similarly, *Gdnf* mRNA can be detected in the metanephric mesenchyme (MM) and not in the ureteric bud (UB) in the developing kidney at E14.5. Scale bars: A, 1.5 mm; B, 5 μm; M, 1 mm; L, 70 μm (left) and 100 μm (right). Abbreviations: bGHpA, bovine growth hormone polyadenylation signal; bp, base pairs; CDS, coding sequence; Cp, quantification cycle; dSTR, dorsal striatum; E, embryonic day; F, FRT sites for Flp recombinase; IHC, immunohistochemistry; kb, kilo-base pairs; m, months; MM, metanephric mesenchyme; P, postnatal day; PV, parvalbumin; *puΔtk*, a cassette with PGK promoter encoding for fusion protein between puromycin N-acetyltransferase and a truncated version of herpes simplex virus type 1 thymidine kinase (TK) with 3’ bovine growth hormone polyadenylation signal; UB, ureteric bud. *P< 0.05, **P< 0.01, and ***P< 0.001; error bars indicate SEM.
(TIF)

S3 Fig Analysis of dopamine system development and function in GDNF hypermorphic mice. **(A-B)** HPLC analysis of DA metabolites DOPAC (A) and HVA (B) in P7.5 rostral brain. N = 5–8 mice/group. **(C-D)** HPLC analysis of DA metabolites DOPAC (C) and HVA (D) in the dSTR at 2.5–3 months of age. N = 11 for *Gdnf* ^*wt/wt*^, 8 for *Gdnf* ^*wt/hyper*^; P = 0.00521 for DOPAC and P = 0.0569 for HVA. **(E)** HPLC analysis of DA levels in the dSTR of *Gdnf 3’UTR* ^*rest/rest*^ and *Gdnf* ^*wt/wt*^ mice; N = 6 mice/group. **(F)** Representative images of DAT immunostaining in the dSTR, N = 7–9 mice/group. **(G-H)** Representative images of TH immunostaining in the dSTR (G) and midbrain (H). In total, 8 mice were analyzed from each genotype. **(I)** Average striatal area size measured from DAT+ sections. (N = 9 for *Gdnf* ^*wt/wt*^, 7 for *Gdnf* ^*wt/hyper*^ **(J)** Representative recording trace of DA events stimulated by single electrical pulses at 2 min intervals, by paired stimulations at shown intervals, and by a burst of 5 stimuli at 20 Hz. **(K-O)** Rise and decay parameters of the first five DA events evoked by single-pulses. N = 5–7 mice/group with 1–3 striatal slices/mouse analyzed. **(K)** DA rise slope is steeper in the striata of *Gdnf* ^wt/hyper^ mice [two-way repeated measures ANOVA, F (1,29) = 11.47, P = 0.0021], indicating that more dopamine is released from the terminals during the rise phase. **(L)** T ½ times, i.e., the width of the event at half of the maximum. **(M)** Rise time, i.e. the time for DA events to rise from baseline to the maximum. **(N)** The fall time of the DA events. **(O)** The exponential decay time constant of the DA transients. Abbreviations: DA, dopamine; DOPAC, 3,4-dihydroxyphenylacetic acid; dSTR, dorsal striatum; HPLC, high performance liquid chromatography; HVA, homovanillic acid; IHC, immunohistochemistry; m, months; P, postnatal day. Scale bars: F, 10 μm; G, 75 μm; in inset 1 mm; H, 0.5 mm; in inset 30 μm.
(TIF)

S4 Fig Analysis of DAT and TH protein levels in GDNF hypermorphic mice and identification of *Gdnf* regulating miR-s. **(A-B).** Total DAT protein levels (A) and plasma membrane associated DAT levels (B) at P7.5, measured by western blotting. N = 4 mice/group. **(C)** DAT protein levels in the dSTR and SNpc at 2.5-3m, measured by western blotting. Gapdh or α-tubulin was used to normalize loading, as indicated in the figure. N = 5 mice/group. **(D)** OD of striatal TH+ fibers at P7.5, reflecting striatal TH levels and the density of striatal dopaminergic innervation at the macroscopic level. N = 7 mice/group. **(E)** TH protein levels in the dSTR at P7.5, measured by western blotting. Gapdh was used to normalize loading. N = 4 mice/group. **(F)** OD of striatal TH+ fibers at 2.5–3 months of age. N = 10–11 mice/group. **(G)** TH protein levels in the dSTR at 2.5–3 months of age, measured by western blotting. Gapdh was used to normalize loading. N = 5 mice/group. **(H)** Luciferase expression from Firefly-*Gdnf* 3’UTR construct in HEK293 cells after co-transfection with constructs encoding RBPs. *Renilla* luciferase expression from a separate plasmid was used for normalization and GFP was used as a negative control. N = 3 experiments with 3 biological repeats each. **(I)** The number of predicted miR binding sites (ddG<0) in *Gdnf* 3’UTR, puΔtk and SV40 late pA signal, analyzed with PITA software [4]. **(J)** The number of predicted strong binding sites for *Gdnf*-specific miRs (ddG<-10) in *Gdnf* 3’UTR, puΔtk and SV40 late pA signal, analyzed with PITA software [4]. Selection of *Gdnf*-specific miRs was based on TargetScan analysis using the most stringent conditions. **(K)** Luciferase expression from *Firefly-puΔtk-Gdnf 3’UTR* construct in HEK293 cells after co-transfection with *Gdnf*-regulating pre-miRs. Note that miRs that inhibit luciferase expression via the native *Gdnf* 3’UTR (Fig 5B) do not suppress the expression from reporter construct containing the *Firefly-puΔtk-Gdnf 3’UTR* cassette. N = 3 experiments with 3–5 biological repeats. **(L)** Luciferase activity in an ATP-based survival assay in U87 cells after co-transfection with pre-miRs. Note that the viability of U87 cells is not affected by the overexpression of pre-miRs, compared to scrambled control pre-miRs. N = 3 experiments with 3 replicates/experiment. **(M)** *Gdnf* mRNA levels in HEK293 cells after transfection with pAAV constructs encoding shRNAs against miR-9, miR-96 and miR-146. A number of shRNA-coding plasmids from two different producers, Signagen (left panel) and Vector Biosystems (right panel), were tested. N = 5 experiments with 2 repeats (all constructs from Signagen) and N = 1–2 experiments (constructs from Vector Biosystems). $ indicates that the constructs were tested 2 times with 2 repeats. The rest of the constructs from Vector Biosystems were tested once with 2 repeats. siRNA against Dicer was included as a positive control. N = 5 experiments with 2 repeats. Abbreviations: AAV, adeno-associated virus; RBP, RNA-binding protein; GFP, green fluorescent protein; AUF1, AU-rich element binding protein 1; ELAVL1, embryonic lethal, abnormal vision-like protein 1; TTP, tristetraprolin; *puΔtk*, a cassette with PGK promoter encoding for fusion protein between puromycin N-acetyltransferase and a truncated version of herpes simplex virus type 1 thymidine kinase (TK) with 3’ bovine growth hormone polyadenylation signal; scr, scrambled control; pA, polyadenylation signal. *P<0.05, **P<0.01, and ***P<0.001; error bars indicate SEM.
(TIF)
