# Correction: GDNF Overexpression from the Native Locus Reveals its Role in the Nigrostriatal Dopaminergic System Function

**DOI:** 10.1371/journal.pgen.1005808

**Published:** 2016-01-11

**Authors:** Anmol Kumar, Jaakko Kopra, Kärt Varendi, Lauriina L. Porokuokka, Anne Panhelainen, Satu Kuure, Pepin Marshall, Nina Karalija, Mari-Anne Härma, Carolina Vilenius, Kersti Lilleväli, Triin Tekko, Jelena Mijatovic, Nita Pulkkinen, Madis Jakobson, Maili Jakobson, Roxana Ola, Erik Palm, Maria Lindahl, Ingrid Strömberg, Vootele Võikar, T. Petteri Piepponen, Mart Saarma, Jaan-Olle Andressoo

The y-axis values in Fig 4M are incorrect. Please view the correct figure here.

**Fig 4:**
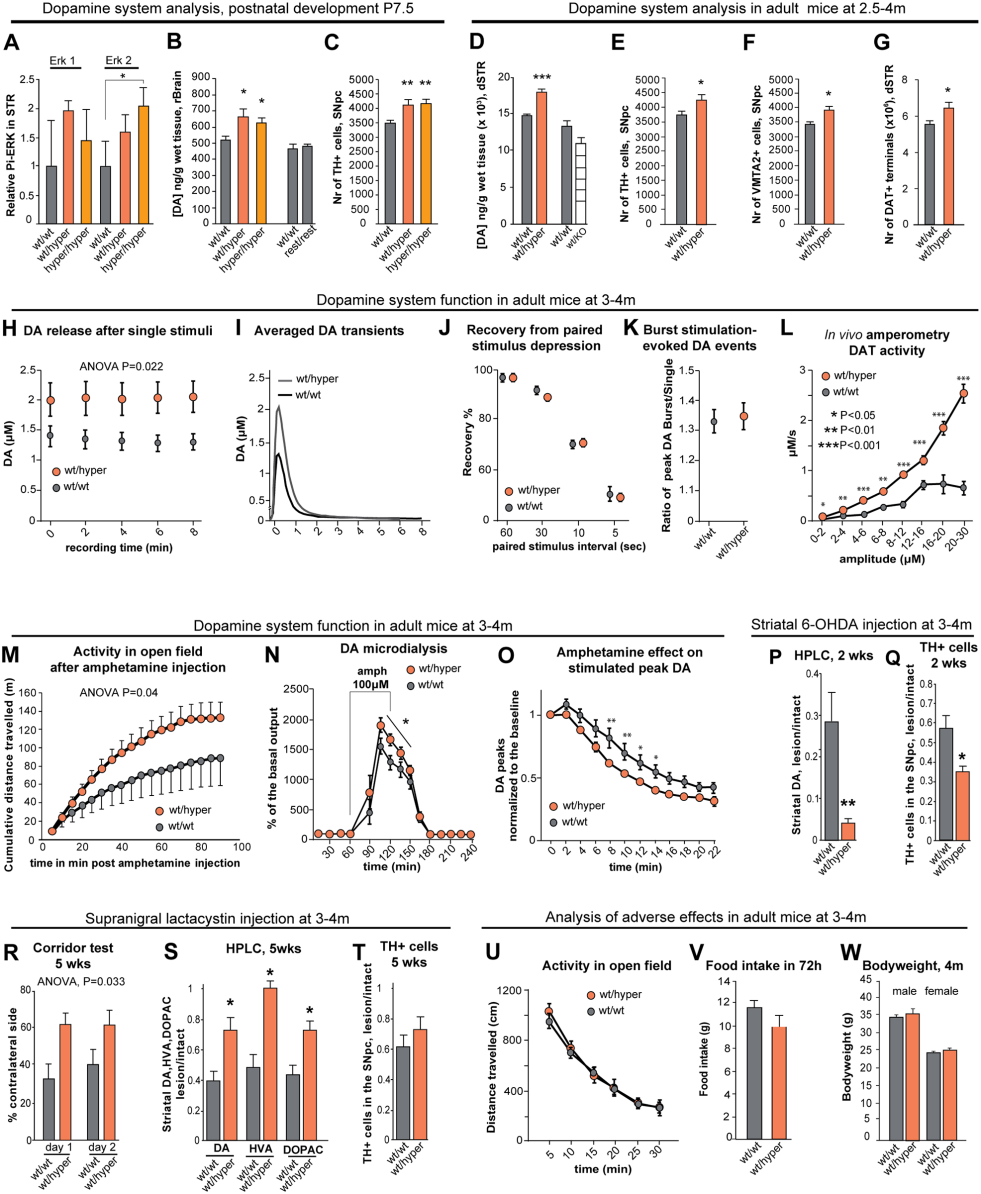
Increased endogenous GDNF expression affects the development and function of the nigrostriatal dopaminergic system. (A) Levels of phosphorylated ERK2 at P7.5 in the striatum of *Gdnf*^*wt/wt*^, *Gdnf*^*wt/hyper*^ and *Gdnf*^*hyper/hyper*^ mice. N = 5 mice/group; ERK was used for normalization. (B) HPLC analysis of DA levels in the rostral brain; N = 5–8 mice/group (F = 7.44, P = 0.016). (C) Quantification of tyrosine hydroxylase (TH)-positive (a marker of DA neurons) cells in the SNpc; N = 6–8 mice/group (F = 7.44, P = 0.0048). (D) HPLC analysis of DA levels in the dSTR; N = 11 for *Gdnf*^*wt/wt*^, 8 for *Gdnf*^*wt/hyper*^ mice/group (P = 0.000164). HPLC analysis of DA levels in the dorsal striatum of *Gdnf 3’UTR*^*wt/wt*^ and *Gdnf*^*wt/KO*^ mice; N = 6 mice/group. (E-F) The number of TH-positive (E; N = 8 *Gdnf*^wt/wt^, N = 7 *Gdnf*^wt/hyper^; P = 0.025) and VMAT2-positive neurons (F; N = 7 *Gdnf*^wt/wt^, N = 7 *Gdnf*^wt/hyper^; P = 0.016) in the SNpc. (G) The number of DAT+ varicosities (N = 9 *Gdnf*^wt/wt^, N = 7 *Gdnf*^wt/hyper^; P = 0.042) in the dSTR. (H-K) Cyclic voltammetry analysis of acute striatal slices (see also S3J Fig); N = 5–7 mice/group with 1–3 slices per mouse. (H) DA release in response to electrical stimulation [two-way repeated measures ANOVA, F (1,29) = 5.866]; (I) Averaged traces of DA events. (J) Short-term depression of striatal DA release after prior DA exocytosis, shown as percent of the first DA release. (K) The ratio of DA release after a single stimulus and after a 5 pulse burst at 20Hz. (L) *In vivo* amperometry following intrastriatal DA injection reveals that dopamine transporter (DAT) activity in *Gdnf*^*wt/hyper*^ mice is dependent on the concentration of DA; N = 4 mice/group (F = 47.931). (M) Locomotor activity after an injection of amphetamine (1 mg/kg, i.p.); N = 9–10 mice/group (F = 4.386, P = 0.04). (N) *In vivo* microdialysis analysis of extracellular striatal DA levels; amphetamine was applied as indicated by the horizontal bar; N = 9 mice/group. (O) Cyclic voltammetry analysis shows that amphetamine (5 μM) depletes stimulated DA release faster in the striata of *Gdnf*^*wt/hyper*^ mice compared to *Gdnf*^*wt/wt*^ mice; two-way repeated-measures ANOVA reveals an effect of time (P<0.0001) and genotype (P = 0.031), as well as an interaction between time and genotype (P = 0.049); N = 6 mice/group with 1–3 slices per mouse. (P-Q) Analysis of a 6-OHDA induced PD model. (P) Quantification of DA in the dSTR 2 weeks after striatal 6-OHDA injection, relative to the intact side (N = 12 *Gdnf*^wt/wt^; N = 10 *Gdnf*^wt/hyper^), (F = 40.62, P = 0.00549, Students t-test). The intact and lesioned side differed significantly (P = 2.71×10^−15^). (Q) Quantification of TH-positive neurons in the SNpc 2 weeks after striatal 6-OHDA injection, relative to the intact side, (F = 7.04, P = 0.0143, Students t-test). The intact and lesioned side differed significantly (P = 3.00×10^−11^). (R-T) Analysis of a lactacystin-induced PD model. (R) The percentage of sugar pellet retrievals from the contralateral side in the corridor test; N = 5–7 mice/group (F = 6.087, P = 0.033). (S) Quantification of DA, DOPAC, and HVA in the dSTR 5 weeks after supranigral lactacystin injection, relative to the intact side; N = 5 *Gdnf*^*wt/wt*^, N = 7 *Gdnf*^*wt/hyper*^; P = 0.046 for DA, P = 0.015 for DOPAC, P = 0.011 for HVA. The intact and lesioned side differed significantly; P = 0.00016 for DA, P = 0.015 for DOPAC, P = 0.010 for HVA. (T) Quantification of TH-positive neurons in the SNpc 5 weeks after lactacystin injection, relative to the intact side; N = 4 *Gdnf*^*wt/wt*^, N = 7 *Gdnf*^*wt/hyper*^; P = 0.236. The intact and lesioned side differed significantly (P = 0.00029). (U-W) Evaluation of side effects associated with intracranial ectopic GDNF expression. (U) Spontaneous locomotor activity in an open field; N = 31–34 mice/group. (V) Food intake by adult mice during a 72-hour period; N = 10 mice/group. (W) Body weight of adult mice; N = 9–34 mice/group. Abbreviations: DA, dopamine; DOPAC, 3,4-dihydroxyphenylacetic acid; HVA, homovanillic acid; dSTR, dorsal striatum; SNpc, substantia nigra pars compacta.
