# Supplementary material for: GDNF Overexpression from the Native Locus Reveals its Role in the Nigrostriatal Dopaminergic System Function
Source: PLoS Genet. 2015 Dec 17;11(12):e1005710. doi: 10.1371/journal.pgen.1005710 (PMC4682981; doi:10.1371/journal.pgen.1005710)
Supplement: S1 Materials and Methods — (DOCX) [file pgen.1005710.s001.docx]

**Supporting Materials and Methods**

**Cell culture**

Human embryonic kidney 293 (HEK-293) cells and human glioblastoma-astrocytoma epithelial-like cell line U-87 MG were purchased from ATCC and cultured at 5% CO_2_, 37 °C in Dulbecco’s Modified Eagle Medium (DMEM, Invitrogen/Gibco) supplemented with 10% Fetal Bovine Serum (FBS, HyClone) and 100 µg/ml Normocin (InvivoGen), if not indicated otherwise. Cells were not allowed to reach confluency beyond 70% at any point during culturing and were split one day before plating for an experiment, unless indicated otherwise.

**Luciferase reporter assay**

PAC clone RP21-583-K20 containing mouse *Gdnf* gene was identified from RPCI 129S6/SvEvTac mouse genomic library using routine radioactive DNA hybridization screening with a probe complementary to *Gdnf* exon 3 and was obtained from CHORI. 2.8 kb mouse *Gdnf* 3’UTR and *puΔtk-Gdnf 3’UTR* cassette were cloned into *XbaI* site as indicated on S Fig 1A in Dual-Luciferase Reporter Assay System vectors (E6911 and E6681, Promega). Constructs were verified by sequencing. Actinomycin D (Sigma) was used at 2 µg/ml. cDNAs encoding for indicated human RBPs in pDEST40 vector (Invitrogen) were obtained from ORFeome Collaboration, generated by Genome Biology Unit cloning service (Biocenter Finland, University of Helsinki) and verified by sequencing. Mutations in *Gdnf* 3’UTR in miR-9, miR-133, miR-146 and miR-96 miR seed sequences were introduced with PCR (see Primers) and verified by sequencing. Mutant sequences were analyzed with PITA software (<http://genie.weizmann.ac.il/>) to ensure that the mutations would not generate new binding sites for any miRs under study. HEK-293 cells (15,000 cells/well) were seeded to a 96-well plate pre-coated with 0.1% gelatin. Reporter plasmids were transfected along with pre-miRs (Ambion) using Lipofectamine 2000 Transfection Reagent (Invitrogen), according to manufacturer’s recommendations. Transfection efficiency was estimated using Cy3- or FAM-labeled negative controls and was shown to be 95% for pre-miRs. The medium was replaced 3–4 hours after the transfection and luciferase expression was measured 24 hours later using Dual-Luciferase Reporter Assay System (Promega) with GlomaX 20/20 luminometer (Promega), as recommended by the manufacturer.

**Analysis of GDNF mRNA and protein levels in U-87 MG cells**

U-87 MG cells were plated on 12-well plates at a density of 100,000 cells/well, and transfected with pre-miRs (Ambion) 24h later using Lipofectamine 2000 (Invitrogen), as recommended by the manufacturer. For RNA isolation, the medium was replaced with fresh cell culture medium after 3–4 hours. Cells were washed with PBS and lysed in TRI Reagent (Molecular Research Center, Inc.) after 48 h for total RNA isolation. *GDNF* mRNA levels were measured as described below in quantitative PCR (qPCR) section.

For protein isolation, the medium was replaced with serum-free OptiMEM (Invitrogen/Gibco) supplemented with 0.5% bovine serum albumin (BSA, Sigma), 3–4 hours after the transfection. The plate was incubated at 8% CO_2_, 37 °C for 48 h as described in[[1](#_ENREF_1)]. GDNF levels were measured from the culture medium using GDNF Emax ImmunoAssay System (Promega), according to the manufacturer’s protocol.

**Analysis of GDNF protein levels in primary cortical neurons**

Cortical neurons were isolated from Dicer-1^fl/fl^ mice [35] on embryonic day 15 (E15) and cultivated as described in [[2](#_ENREF_2)]. Adenovirus encoding either Cre recombinase or green fluorescent protein (GFP) was introduced as described in [[3](#_ENREF_3)] and GDNF protein levels were measured from the culture medium using GDNF Emax ImmunoAssay System (Promega) according to the manufacturer’s protocol.

**RNAi experiments**

HEK-293 cells were seeded to 12-well plates at a density of 200,000 cells/well and incubated for 24 h to reach 80–90% confluency. The cells were transfected with five candidate constructs/miR (S Fig 4M); AAV-CAG-GFP-mmu-miR9-shRNA, AAV-CAG-GFP-mmu-miR96-shRNA, AAV-CAG-GFP-mmu-miR146-shRNA and AAV-CAG-GFP-scramble-shRNA (Vector Biosystems) and one candidate construct/miR AAV-U6-shmir9-GFP AAV-U6-shmir96-GFP, AAV-U6-shmir146-GFP and AAV-U6s-Scramble-shRNA-GFP (Signagen), or SignalSilence Dicer siRNA II (Cell Signaling) (S Fig 4M). Transfection medium was replaced with growth medium (GM) 4 h after the transfection, and the cells were cultured for 72 h, with GM replaced at 48h. The cells were homogenized with TRI Reagent (Molecular Research Center, Inc.) and total RNA was isolated as recommended by the manufacturer. All experiments were performed with two technical repeats and experiments were repeated 2–5 times.

***In vitro* transcription and Gdnf sense and anti-sense transcript analysis**

1µg of pBluescript containing mouse *Gdnf* exons was linearized with restriction endonuclease Xho l (Thermo Scientific) or Hind III (Thermo Scientific), as recommended by the manufacturer, to enable transcription of either antisense or sense *Gdnf* RNA, respectively. Linearized product was purified with NucleoSpin Gel and PCR Clean-up (Macherey-Nagel) and dissolved in water. For *in vitro* transcription of antisense and sense RNA, the purified plasmid product was transcribed with T7 or T3 RNA polymerase (Promega), respectively, as recommended by the manufacturer. RNA was treated with RQ1 RNase-free DNase (Promega) prior to reverse transcription with RevertAid Premium Reverse Transcriptase (Thermo Scientific). cDNA for *Gdnf* sense (coding) strand was synthesized using random hexamer primers (Thermo Scientific) and cDNA for *Gdnf* antisense strand was synthesized using a strand-specific primer 5’-AGGTCACCAGATAAACAAGC-3’.

**Northern blotting**

Total RNA from cells or mouse tissues was isolated using TRI Reagent (Molecular Research Center, Inc.). Northern blotting was performed using standard procedures. Briefly, RNA was electrophoresed in 1% denaturing agarose gel, transferred to nylon membrane, and specific RNAs were detected using indicated probes with DIG-based nucleotide detection system (Roche)*.*

**Cell survival assay**

Survival of U-87 cells was assayed using CellTiter-Glo Luminescent Cell Viability Assay (Promega) according to manufacturer’s protocol.

**Public databases**

Evolutionary conservation of miR seed sequences in *Gdnf* 3’UTR was evaluated using the most stringent conditions of TargetScan (http://www.targetscan.org/). BLAST was used to assess the overall sequence conservation of *Gdnf* 3’UTR. miR-96 and miR-9 were identified to interact with *Gdnf* 3’UTR in two genome wide screens: i) miR CLIP-seq dataset using Argonaute cross-linked immunoprecipitation followed by RNA sequencing [34], and ii) microarray analyses following miR transfection [34] <http://servers.binf.ku.dk/antar/>. Prediction of AU-rich elements as potential binding sites for RNA-binding proteins in *Gdnf* 3’UTR was performed using AREsite (http://rna.tbi.univie.ac.at/cgi-bin/AREsite.cgi). The number of potential miR binding sites in *Gdnf* 3’UTR, puΔtk cassette [12], bovine growth hormone polyadenylation signal (bGHpA) and SV40 late pA was performed using PITA [[4](#_ENREF_4)].

**Generation and genotyping of GDNF hypermorphic animals**

*Gdnf puΔtk-Gdnf 3’UTR* (*Gdnf^hyper^*) allele was generated with routine methods using IB10 ES cells. Briefly, 5667bp 5’ homologous arm spanning the second intron of the *Gdnf* gene, 6055bp 3’ homologous arm and *Gdnf* exon 3, including the stop codon were amplified with PCR from *Gdnf*-containing PAC (RP21-583-K20, CHORI) and cloned into *Pme*I, *Not*I and *Hind*III sites in pFlexible [[5](#_ENREF_5)], respectively, to generate *Gdnf* targeted allele (S Fig 1G)**.** ES clones that had undergone homologous recombination (17% of 120 clones) were first identified using PCR, followed by verification by Southern blotting with a 3’ probe located outside the homologous arms (S Fig 1G-H). ES clones G26 and G45 were karyotyped and used for morual aggregations to generate the *Gdnf^hyper^* mouse line. Routine genotyping of the mice (S Fig 1I) was performed using PCR primers indicated on S Fig 1G. Mice were maintained in 129Ola/ICR/C57bl6 mixed genetic background, housed in 12/12 light/dark cycle with “lights-on” at 06:00, at ambient temperature of 20–22 °C, 2–5 animals per cage with *ad libitum* access to standard chow and water. All animal experiments were authorized by the national Animal Experiment Board of Finland.

**Tissue dissection**

Except for the neurotransmitter levels analysis (see below), mice were euthanized with CO_2_, followed by decapitation. The tissues were quickly isolated and cooled in ice-cold saline. The brains were placed on an ice-cold brain matrix (Stoelting) for further dissection of the indicated brain areas. For isolation of the rostral brain at P7.5, the brains were cut in half with a razor blade at -0.2 mm from bregma and the dorsal part of the brain was collected. For qPCR or Western blotting (WB), the tissues were immediately frozen and stored at -80°C until assayed.

**Analysis of kidney development**

Embryos and tissues of indicated stages were collected and fixed overnight with 4% paraformaldehyde (PFA). Processing for paraffin sections was performed according to standard procedures, utilizing an automatic tissue processor (Leica ASP 200). Hematoxylin-eosin and whole mount immunofluorescence staining were performed as previously described [[6](#_ENREF_6)]. Briefly, E11.5 whole-mount urogenital blocks (UGBs) were cultured for 3h in DMEM/F-12, Glutamax supplement (Life Technologies), containing 10% FBS and penicillin-streptomycin, and fixed with 10% methanol for 10 min. E13.5 UGBs were fixed with 10% methanol without prior culturing. The tissues were then stained with calbindin or E-cadherin and imaged with Zeiss Imager M2 Axio equipped with Zeiss AxioCam HRm camera and Axio Vision 4 software, or Leica stereomicroscope.

**Serum urea and creatinine**

Serum urea and creatinine levels were measured with standard kits (BioAssay Sytems).

**Estimation of monoamines and their metabolites**

Dopamine and its metabolites were analyzed as described in [[7](#_ENREF_7)] using HPLC with electrochemical detection.

**Immunohistochemistry (IHC) for light microscopy**

The mice were anesthetized with sodium pentobarbital (100 mg/kg, i.p.) and intracardially perfused with PBS followed by 4% PFA in 0.1 M phosphate buffer, pH 7.4. The brains were fixed in 4% PFA for 4 h, and stored in phosphate buffer containing 20% sucrose at 4 °C. In an alternative ’light‘ perfusion method, brains were cooled after PBS perfusion and dorsal striatum was dissected from the rostral part of the brain, while the posterior part containing the midbrain was fixed overnight in 4% PFA for IHC. The latter method was used to reduce the number of experimental animals in experiments with PD model. Coronal striatal (30 µm) and nigral (40 µm) sections were cut using microtome and stored in serial order at -20°C until processed for immunostaining.

*TH, DAT and VMAT2 immunohistochemistry.* Staining of freely floating sections was performed using standard immunohistochemical procedures, using the following antibodies: rabbit-anti-TH (1:2000; AB 152, Millipore), rat-anti-DAT (1:3000; MAB369, Millipore), goat-anti-VMAT2 (1:4000; ab87594, Abcam), biotinylated goat-anti-rabbit (1:200; BA1000, Vector Laboratories), biotinylated rabbit-anti-rat (1:200; BA4000, Vector) and biotinylated horse-anti-goat (1:200; PI-9500, Vector Laboratories). Vectastain Elite ABC peroxidase kit (Vector Laboratories) was used for visualization. See [43] for further details.

**Stereological analysis of TH- and VMAT2-positive cells and DAT-positive varicosities.** The number of TH- and VMAT2-positive neurons in the substantia nigra pars compacta (SNpc) was assessed by a person blinded to the identity of the samples. Briefly, cells positive for TH or VMAT2 were counted at the medial region of the SNpc, around the medial terminal nucleus. From each adult animal, every third section between levels −3.08 and −3.28 mm from the bregma was selected (3 sections per animal). From each P7.5 animal, every second section between levels −2.92 and −3.16 mm from the bregma was selected (3 sections per animal). StereoInvestigator (MBF Bioscience) was used to outline the SNpc, and positively stained cells were counted within the defined outlines according to optical dissector rules[[8](#_ENREF_8)]. Cells were counted at regular predetermined intervals (x = 100 μm; y = 80 μm) within the counting frame (60 μm × 60 μm) superimposed on the image using a 60× oil objective [Olympus BX51 (Olympus Optical) equipped with an Optronics camera]. The counting frame positions within the SNpc were randomized by the software. The coefficient of error (CE) was calculated as an estimate of precision and values <0.1 were accepted. Failure in staining or perfusion resulting in spoiled sections was an exclusion criterion. Please see [43] for further details.

The number of DAT-immunoreactive varicosities in the dorsal 500 µm of striatum was estimated as described in [37, 43] using the same system as for SNpc cell counts. Every sixth striatal section from planes 1.1 and 0.38 from bregma (total four sections of each animal) was analyzed under 100x oil objective. Counts of DAT positive varicosities were made at regular, predetermined intervals (x = 170 µm; y = 170µm) with an unbiased counting frame of 5 µm x 4 µm. DAT-positive terminals were identified as round axonal swellings and used as counting units by an experimenter blinded to genotypes The CE was calculated as an estimate of precision, and values <0.1 were accepted. Failure in staining or perfusion resulting in spoiled sections was an exclusion criterion.

**Striatal size.** Striatal area size was estimated from the same four sections that were used for counting DAT-positive varicosities (see above) using the stereoinvestigator software (MBF Bioscience) and 4x objective. To determine the striatal area size a contour was drawn around the DAT-immunoreatice area using nucleus accumbens [as depicted in the atlas of Franklin and Paxinos (1997)] as the ventral border. The average area of the four sections was taken as the final measure.

**Striatal densitometry.** The optical density (OD) was analyzed by a person blinded to the identity of the samples using Optronics digital camera and Image-Pro Plus software (Version 3.0.1; Media Cybernetics). Three striatal sections from each animal were analyzed and averaged. The nonspecific background correction in each section was done by subtracting the OD value of the corpus callosum from the striatal OD value of the same section. Failure in staining or perfusion resulting in spoiled sections was an exclusion criterion. Please see [43] for further details.

**Amphetamine-induced locomotor activity**

Mice were individually placed in an open-field activity monitor (MED Associates), and habituated for about 15 min before the injection of D-amphetamine-sulphate (1 mg/kg, i.p; Division of Pharmaceutical Chemistry, Faculty of Pharmacy, University of Helsinki, Finland). Locomotor activity was monitored for 60 min. Animals that gave no locomotor response to amphetamine (<2000 cm in 60 min) were excluded from the experiment.

**Lactacystin model of Parkinson’s disease**

4 µg of lactacystin (AG Scientific) in 4 µl of PBS was injected to 3 month old male mice just above the SN at the following stereotaxic coordinates: antero-posterior (AP) -3.3 mm; medio-lateral (ML) -1.2 mm and dorso-ventral (DV) -4.6 mm. The animals were subjected to corridor test [[9](#_ENREF_9)] 5 weeks after the injection, and sacrificed for tissue isolation and IHC analysis. Analyses were performed by experimenters blinded to the genotype. Exclusion criteria were: histological analysis revealing a needle puncture or other physical damage in the SNpc; or the lesion in terms of reduction in TH-positive cell number in the SN was completely absent indicating failure in LC injection.

**6-OHDA model of Parkinson’s disease**

Right striatum was lesioned with 6-hydroxydopamine as previously described [42]. Briefly, 6-hydroxydopamine (6-OHDA hydrochloride; Sigma, St. Louis, MO) dissolved in 0.02 % ascorbic acid saline solution was injected into the right striatum (AP +0.7; ML -1.8 and DV -2.7) of 3 months old male mice under isoflurane anesthesia (5 µg of 6-OHDA, total volume injected was 2 µl, rate of injection was 0.5 µl/min). In order to protect noradrenergic nerve terminals mice received desipramine 25 mg/kg intraperitoneally (i.p.) (desipramine hydrochloride, Sigma) 30 minutes before the 6-OHDA injections. Two weeks after the injections the animals were sacrificed for tissue isolation and IHC analysis. Analyses were performed by experimenters blinded to the genotype.

**Whole-mount in situ hybridization**

Whole-mount *in situ* hybridization was performed with InSituPro Automate (Intavis) as described previously [[10](#_ENREF_10)] using either a probe spanning all mouse *Gdnf* exons or 525bp in the 3’ end of mouse *Gdnf* 3’UTR. The *in situ* hybridization protocol for the P7.5 and P12.5 brain and spinal cord tissue was modified from the routine as follows. The tissues were fixed in 4% PFA at 4 °C for 3-7 days, incubated overnight with 20% sucrose in 4% PFA at 4 °C. Freely floating cryosections (40 μm) were washed for 20 min in ice-cold PBS containing 0.25% TritonX-100, followed by 5 min incubation with 5x SSC (pH5) at RT. Prehybridization was carried out for 2 h, followed by overnight hybridization with 1 μg/ml Dig-labelled RNA probe at 65 °C in 50% formamide, 5x SSC (pH5), 2% blocking reagent (Roche). Posthybridization washes were as follows: i) 50% formamide, 5x SSC (pH5), 1% SDS for 30 min at 65°C; ii) 50% formamide, 2x SSC (pH5) for 30 min at 60°C; followed by three washes with TBST. BM Purple AP Substrate (Roche) was used as substrate. All steps were performed with shaking. The stained sections were transferred onto slides in 0.5% gelatin, air dried and mounted with Pertex (Histolab).

**RNAscope analysis**

RNAscope [20] probes detecting *Gdnf* (red) and *Parvalbumin (PV*, blue) mRNA were custom made by Advanced Cell Diagnostics and were hybridized to slices from E14.5 kidney, and cerebellum and striatum of 3 month old mice, according to manufacturer’s recommendations. *Gdnf, PV* and double positive striatal cells were counted by a person blinded to the identity of the samples.

**Fast-scan cyclic voltammetry**

13–15 week old mice were decapitated and 300 µm coronal slices that contained cortex and striatum were cut on a vibrating microtome (7000 smz-2, Campden Instruments) in ice-cold cutting saline containing 125 mM NaCl, 2.5 mM KCl, 26 mM NaHCO_3_, 0.3 mM KH_2_PO_4_, 3.3 mM MgSO_4_, 0.8 mM NaH_2_PO_4_, and 10 mM glucose. Slices were allowed to recover in holding chamber for 1–2 hours at 35 °C, in oxygen-bubbled (95% O_2_, 5% CO_2_) recording saline containing 125 mM NaCl, 2.5 mM KCl, 26 mM NaHCO_3_, 0.3 mM KH_2_PO_4_, 2.4 mM CaCl_2_, 1.3 mM MgSO_4_, 0.8 mM NaH_2_PO_4_, and 10 mM glucose. In the recording chamber the slices were continuously perfused with 35 °C oxygen-bubbled recording saline. In amphetamine (d-amphetamine hemi-sulfate, Sigma-Aldrich) experiments the drug was added to the perfusion (5 uM) after a stable baseline of stimulated transient peaks was reached. Fast-scan cyclic voltammetry recordings were performed with cylinder 5 μm carbon fiber electrodes positioned at the dorsal striatum ~50 μm below the exposed surface. Striatal slices were electrically stimulated using a bipolar stainless steel electrode placed at a distance of ~100 μm from the recording electrode. Square pulses of 0.4 ms duration were produced by an Iso-Flex stimulus isolator triggered by a Master-8 pulse generator (A.M.P.I.). Stimulus magnitude was selected by plotting a current–response curve and selecting the minimum value that reliably produced the maximal response. Triangular voltage ramps from −450 mV holding potential to +900 mV over 9 ms (scan rate of 300 mV/ms) were applied to the carbon fiber electrode at 100 ms intervals. Current was recorded with an Axopatch 200B amplifier (Molecular Devices) filtered with 5 kHz low-pass Bessel filter and digitized at 40 kHz (ITC-18 board; InstruTech). Triangular wave generation and data acquisition were controlled and the recorded transients were characterized by a computer routine in IGOR Pro (WaveMetrics) [[11](#_ENREF_11),[12](#_ENREF_12)]. Background-subtracted cyclic voltammograms obtained with 1 uM of dopamine solution (Dopamine-HCl, Sigma-Aldrich) were used to calibrate the electrodes and to identify dopamine. The DA terminals were stimulated either with single electrical pulses at 2 min intervals; paired stimulations at fixed intervals of 60, 30, 10 and 5 sec to study paired stimulation depression or by a burst stimulation of 5 pulses at 20 Hz, to study the release probability of the terminals[[13](#_ENREF_13),[14](#_ENREF_14)].

***In vivo* chronoamperometry**

*In vivo* chronoamperometry with second-by-second quantitative detection of dopamine levels was performed with the Fast Analytical Sensing Technology (FAST-16) system (Quanteon)12 using single carbon fiber electrodes (Quanteon) coated with Nafion (Sigma). The electrodes were calibrated in phosphate buffered saline (0.05 M PBS, pH=7.4), and 20 mM ascorbic acid and dopamine (2 mM) were added during the calibration procedure. Only electrodes with selectivity of more than 200:1 for DA over ascorbic acid, a limit of detection below 0.05 µM, and linear response to DA (R2 >0.995) were used. Following calibration, the electrode was mounted in parallel with a micropipette used for application of dopamine with a distance of 130-160 µm between the tips. Mice were anesthetized with urethane (1.7–1.9 g/kg, i.p.; Sigma) and fixed in a stereotaxic frame on a heating pad. An incision was made in the scalp and the bone overlying the striatum was removed. An additional single hole was made caudally for implantation of an Ag/AgCl reference electrode. The electrode/micropipette-assembly was lowered into the striatum, using a microdrive. Recordings were performed at two distinct rostrocaudal striatal tracks in each hemisphere, at stereotaxic coordinates AP +0.3 or +1.0 mm; ML ±1.8 mm, calculated from bregma level. At each recording site, data was collected from three depths below the dura: at -2.0, -2.5, and -3.0 mm. The ejected volume (25–75 nl) was monitored using a scale fitted in the ocular of an operation microscope. Dopamine (200 µM, in saline containing 20 µM ascorbic acid) was locally applied to evaluate dopamine clearance.

During recordings, a square wave potential of 0.55 and 0 V (against an Ag/AgCl reference electrode) was applied over the electrode at a frequency of 5 Hz. Current produced from the oxidation and reduction reactions were integrated, giving an average signal per second for each reaction. Intrastriatally injected DA (200 µM) produced a range of amplitudes at each stereotaxic coordinate. The data points were pooled for analysis and DA peaks (in μM) were separated into amplitude bins and plotted against uptake rate, μM/s; calculated using Michaelis-Menten first-order rate constant, k1.

## *In vivo* microdialysis

A microdialysis guide cannula (MAB 4.1, AgnTho’s AB) was inserted into the dorsal striatum (AP +0.6 mm; ML +1.8 mm and DV -2.2 mm) of 4-5 month old male mice under isoflurane anaesthesia. Two screws and dental cement (Aqualox) attached the guide cannula into the skull. After at least 4 days of recovery, a microdialysis probe (MAB 4.9.1.Cu; AgnTho’s AB) was inserted into the guide cannula and dialysis was started with Ringer solution (147 mM NaCl, 1.2 mM CaCl_2_, 2.7 mM KCl, 1.0 mM MgCl_2_, and 0.04 mM ascorbic acid) at a flow rate of 2 µl/min. Sample collection started after two hours of stabilization at 15 min intervals. Concentration of dopamine was analyzed with HPLC using electrochemical detection (Coulochem II; ESA, Inc.). The column (Kinetex 2.6u; XB-C18; 50 x 4.6 mm; Phenomenex) was kept at 45 °C with a column heater. The flow rate of mobile phase (0.1 M NaH_2_PO_4_, pH 4, 0.1 mg/ml octanesulphonic acid, 1.0 mM EDTA and 8% methanol) was 1 ml/min. 25 µl of the sample was injected into the chromatographic system with autoinjector (SIL-20AC, Shimadzu). After obtaining a stable baseline (determined as an average of four consecutive samples), the Ringer solution was switched into one containing 100 µM of D-amphetamine (Division of Pharmaceutical Chemistry, Faculty of Pharmacy, University of Helsinki, Finland) for 60 minutes. After the experiment, the animals were decapitated and brains removed and frozen. The correct placement of the probe was histologically confirmed from 90 µm thick coronal brain sections.

**Measurements of GDNF protein levels from tissues**

GDNF protein levels in E18.5 kidney and testis were analyzed using GDNF Emax ImmunoAssay System (Promega, USA) according to the manufacturer’s protocol.

Striatal tissues were isolated on ice and sonicated immediately in ice-cold lysis buffer, pH 7.4 containing 8.1 mM Na_2_PO_4_, 137 mM NaCl, 2.7 mM KCl, 1% Igepal CA-630 (Sigma), 10% glycerol and protease inhibitor cocktail tablet Complete EDTA-free Mini (Roche). Lysate was centrifuged for 5 min at 5000 rpm at 4 °C and supernatant was used directly or aliquoted and snap frozen on dry ice and stored at –80 °C. Aliquots were thawed only once for ELISA analysis. Here and below, total protein concentrations were measured using DC Protein Assay (Bio-Rad), as recommended by the manufacturer, and 100–200 µg of total protein was analyzed in GDNF ELISA. GDNF Emax ImmunoAssay System (Promega) was used as recommended by the manufacture, including acid treatment. Absorbance at 450 nm was recorded with Victor3 plate reader (Wallac-PerkinElmer). Striatal lysate from adult mice lacking GDNF specifically in the brain (*Gdnf^FLOXED/KO^ + Nestin-Cre* mice [10]) was included in every analysis to define the background signal.

**Quantitative PCR (qPCR)**

RNA was treated with RQ1 RNase-free DNAse (Promega) and reverse transcription (RT) reaction was carried out using RevertAid Reverse Transcriptase (Fermentas), as recommended by the manufacturer. qPCR reaction was performed with SYBRgreen (Roche) using the LightCycler 480 Real-Time PCR System (Roche) in 3–4 repeats. β-actin served as a reference gene. Primer pairs used for qPCR are indicated below. For miR expression analysis, cDNA was synthesized with TaqMan MicroRNA Reverse Transcription Kit (Applied Biosystems) using Megaplex RT Primers, Rodent Pool A or B (Applied Biosystems) without preamplification. qPCR reaction was performed using TaqMan Universal Master Mix II, no UNG (Life Technologies) according to manufacturer’s recommendations. miR expression was normalized to sno202. Each sample was run in duplicate.

**Western blotting (WB)**

Dorsal striatum and SN were isolated and homogenized on ice. 20 μg of protein samples were run on sodium dodecyl sulfate-polyacrylamide gel electrophoresis (SDS-PAGE) and blotted to nylon membranes. Blocking was performed in 5% non-fat milk in 0.1% Tween20 in TBS. The following antibodies were used: mouse-anti-TH (1:3000; MAB318, Chemicon), rat-anti-DAT (1:2500; MAB369, Chemicon MAB369), mouse-anti-p-ERK (1:1000; sc7383, Santa Cruz), rabbit anti-Erk (1:1000; sc94, Santa Cruz), mouse-anti-GAPDH (1:10,000; MAB372, Millipore), mouse-anti-α-tubulin (1:30,000; T9026, Sigma), donkey-anti-mouse-HRP (1:3000; P0449, Dako), anti-rabbit-HRP (1:3000; NA9340, GE Healthcare), and biotinylated rabbit-anti-rat (1:1000; BA-4000, Vector Laboratories) and streptavidin-HRP (1:2500; S-911, Molecular Probes). Signal was visualized with Pierce ECL Western Blotting Substrate (Thermo Scientific) and the intensities of the bands were analyzed using ImageJ software.

**Analysis of DAT protein levels on striatal cell surface**

Striata from P7.5 mice were sliced into 350 μm sagittal sections with a vibratome (Vibratome Co.), washed in ice-cold PBS and incubated on ice in PBS for 1h in the absence or presence of cod trypsin (2 U/ml) (Zymetech), followed by snap freezing and subsequent analysis by WB. 20 μg of protein was loaded to SDS-PAGE. WB for DAT was performed as described above.

**Behavioral and CLAMS analysis**

Locomotor activity was tested in open field in three independent cohorts of comparable size (N=10–12 male mice per genotype in each experiment). Experiment was performed by three different experimenters blind to the genotypes. Animals were tested in a randomized manner during the light period of the day between 9:00 am and 4:00 pm. Metabolic monitoring (food intake) was performed as described in detail in [[15-17](#_ENREF_15)] using Comprehensive Lab Animal Monitoring System (CLAMS).

**Primers**

**Mutation primers (mutated nucleotides indicated in bold):**

miR-9 accaaag -> a**aacc**ag (4 nt mutation)
F: aaaccagttctctacaaactttatttttg
R: tgtataacacaaacgaccgag

miR-133 site 1 ggaccaa -> **acgaagc** (7 nt mutation)
F: acgaagcggttcccaggaaatgtttgcc
R: ctttcttcgcaactgtagcag

miR-133 site 2 ggaccaa -> **acgaagc** (7 nt mutation)
F: acgaagcgatggcggaggcagaggca
R: ttatcttccactctgggcaaac

miR-146 site agttctc -> agt**ctct** (4 nt mutation)
F: agtctcttacaaactttatttttgtacaatatc
R: ttggttgtataacacaaacgac

miR-96 site gtgccaaa -> **cgaaagtc** (8 nt mutation)

F: cgaaagtcgtatatgtgctcacaaaatacaaag
R: ctttgaaaagagactttaataaataag

**qPCR primers:***Gdnf*
F: cgctgaccagtgactccaatatgc
R: tgccgcttgtttatctggtgacc

*beta-actin*
F: ccagttcgccatggatgac
R: gagccgttgtcgacgacc

*Analysis of Gdnf-puΔtk fusion transcript in GDNF hypermorphic mice*

F: cgctgaccagtgactccaatatgc

R: tacccgcttccattgctc

**Statistical analysis**

Data from biological repeats, i.e. cDNA or tissue/cell lysates derived from different animals or cell culture dishes was subjected for statistical analysis. In case of technical repeats (qPCR, ELISA), mean values were used in calculations; in luciferase assays technical repeats from each experiment (N=2-5 experiments) were used in calculations. Statistical analysis for pairwise comparisons was performed using Student’s t-test with two tailed distribution using the unequal variance option. For statistical analysis of the qPCR data, average Cp value of the reference gene (obtained from the Absolute Derivative Max function with the Lightcycler 480 Software Release 1.5.0 SP1 software) was subtracted from the Cp value of the gene of interest and the resulting dCt value was used to calculate fold difference relative to the reference gene (2^-dCt^). Data from amperometry was analyzed by one-way ANOVA followed by Bonferroni *post hoc* test. Behavioral data were analyzed using factorial ANOVA design with genotype and cohort as between-subject factors, where appropriate. *Post hoc* analysis after significant ANOVA was carried out using Student-Newman-Keuls test. All numerical results are reported as mean ± standard error of the mean. SPSS (IBM Corp., Armonk NY, USA) or STATISTICA 11 (StatSoft Inc., Tulsa) were used for analysis.

**References**

1. Verity AN, Wyatt TL, Lee W, Hajos B, Baecker PA, et al. (1999) Differential regulation of glial cell line-derived neurotrophic factor (GDNF) expression in human neuroblastoma and glioblastoma cell lines. J Neurosci Res 55: 187-197.

2. Jakobson M, Lintulahti A, Arumae U (2012) mRNA for N-Bak, a neuron-specific BH3-only splice isoform of Bak, escapes nonsense-mediated decay and is translationally repressed in the neurons. Cell Death Dis 3: e269.

3. Anton M, Graham FL (1995) Site-specific recombination mediated by an adenovirus vector expressing the Cre recombinase protein: a molecular switch for control of gene expression. J Virol 69: 4600-4606.

4. Kertesz M, Iovino N, Unnerstall U, Gaul U, Segal E (2007) The role of site accessibility in microRNA target recognition. Nat Genet 39: 1278-1284.

5. van der Weyden L, Adams DJ, Harris LW, Tannahill D, Arends MJ, et al. (2005) Null and conditional Semaphorin 3B alleles using a flexible puro Delta tk LoxP/FRT vector. Genesis 41: 171-178.

6. Chi X, Michos O, Shakya R, Riccio P, Enomoto H, et al. (2009) Ret-dependent cell rearrangements in the Wolffian duct epithelium initiate ureteric bud morphogenesis. Dev Cell 17: 199-209.

7. Valros A, Palander P, Heinonen M, Munsterhjelm C, Brunberg E, et al. (2015) Evidence for a link between tail biting and central monoamine metabolism in pigs (Sus scrofa domestica). Physiol Behav 143: 151-157.

8. Gundersen HJ, Bagger P, Bendtsen TF, Evans SM, Korbo L, et al. (1988) The new stereological tools: disector, fractionator, nucleator and point sampled intercepts and their use in pathological research and diagnosis. APMIS 96: 857-881.

9. Grealish S, Mattsson B, Draxler P, Bjorklund A (2010) Characterisation of behavioural and neurodegenerative changes induced by intranigral 6-hydroxydopamine lesions in a mouse model of Parkinson's disease. Eur J Neurosci 31: 2266-2278.

10. Ola R, Jakobson M, Kvist J, Perala N, Kuure S, et al. (2011) The GDNF target Vsnl1 marks the ureteric tip. J Am Soc Nephrol 22: 274-284.

11. Mosharov EV (2008) Analysis of single-vesicle exocytotic events recorded by amperometry. Methods Mol Biol 440: 315-327.

12. Mosharov EV, Sulzer D (2005) Analysis of exocytotic events recorded by amperometry. Nat Methods 2: 651-658.

13. Cragg SJ (2003) Variable dopamine release probability and short-term plasticity between functional domains of the primate striatum. J Neurosci 23: 4378-4385.

14. Senior SL, Ninkina N, Deacon R, Bannerman D, Buchman VL, et al. (2008) Increased striatal dopamine release and hyperdopaminergic-like behaviour in mice lacking both alpha-synuclein and gamma-synuclein. Eur J Neurosci 27: 947-957.

15. Kulesskaya N, Rauvala H, Voikar V (2011) Evaluation of social and physical enrichment in modulation of behavioural phenotype in C57BL/6J female mice. PLoS One 6: e24755.

16. Voikar V, Polus A, Vasar E, Rauvala H (2005) Long-term individual housing in C57BL/6J and DBA/2 mice: assessment of behavioral consequences. Genes Brain Behav 4: 240-252.

17. Voikar V, Vasar E, Rauvala H (2004) Behavioral alterations induced by repeated testing in C57BL/6J and 129S2/Sv mice: implications for phenotyping screens. Genes Brain Behav 3: 27-38.
