# Supplementary material for: GDNF Overexpression from the Native Locus Reveals its Role in the Nigrostriatal Dopaminergic System Function
Source: PLoS Genet. 2015 Dec 17;11(12):e1005710. doi: 10.1371/journal.pgen.1005710 (PMC4682981; doi:10.1371/journal.pgen.1005710)
Supplement: S1 Table — Gdnf KO animals were obtained by crossing Gdnf wt/hyper animals to Deleter-Cre mice, which results in deletion of GDNF protein coding exon 3 (Fig 1B) [10]. (DOCX) [file pgen.1005710.s002.docx]

**Supporting Table 1. GDNF levels regulate kidney size and number.**

| **Genotype, P7.5** | **Nr of animals analysed** | **Two kidneys** | **One kidney** | **No kidneys** | **Kidney size** |
| --- | --- | --- | --- | --- | --- |
| **Gdnf ^wt/wt^** | 141 | 141 | - | - | normal |
| **Gdnf ^wt/hyper^** | 60 | 60 | - | - | normal or mildly reduced |
| **Gdnf ^hyper/hyper^** | 14 | 14 | - | - | severely reduced |
| **Gdnf ^wt/KO^** | 34 | 26 | 8 | - | normal |
| **Gdnf ^KO/KO^ (E18.5)** | 7 | - | - | 7 | no kidneys |

Gdnf KO animals were obtained by crossing *Gdnf^wt/hyper^* animals to Deleter-Cre mice, which results in deletion of GDNF protein coding exon 3 (Fig. 1B) [10].
