# Supplementary material for: GDNF Overexpression from the Native Locus Reveals its Role in the Nigrostriatal Dopaminergic System Function
Source: PLoS Genet. 2015 Dec 17;11(12):e1005710. doi: 10.1371/journal.pgen.1005710 (PMC4682981; doi:10.1371/journal.pgen.1005710)
Supplement: S2 Table — (DOCX) [file pgen.1005710.s003.docx]

**Supporting Table 2. *Gdnf^hyper/hyper^* mice die before weaning, whereas *Gdnf^wt/hyper^* mice are produced in Mendelian ratios.**

| **Developmental stage** | **Nr of animals analysed** | **Gdnf ^wt/hyper^ expected** | **Gdnf ^wt/hyper^ found** | **Gdnf ^hyper/hyper^ expected** | **Gdnf ^hyper/hyper^ found** |
| --- | --- | --- | --- | --- | --- |
| **E10-E18** | 116 | 58 | 62 | 29 | 26 |
| **P7.5** | 105 | 53 | 60 | 26 | 14 |
| **P18** | 22 | 11 | 14 | 6 | 0 |
| **2-4 months** | 101 | 50 | 53 | 25 | 0 |
