# Supplementary material for: GDNF Overexpression from the Native Locus Reveals its Role in the Nigrostriatal Dopaminergic System Function
Source: PLoS Genet. 2015 Dec 17;11(12):e1005710. doi: 10.1371/journal.pgen.1005710 (PMC4682981; doi:10.1371/journal.pgen.1005710)
Supplement: S3 Table — Correlational analyses of rostral brain dopamine and serum urea levels in individual animals in 10 Gdnf wt/wt and 12 Gdnf wt/hyper animals at P7.5 using the Correlation function in Microsoft Excel. (DOCX) [file pgen.1005710.s004.docx]

**Supporting Table 3. Lack of correlation between serum urea and rostral brain dopamine levels in individual *Gdnf^wt/hyper^* mice.** Correlational analyses of rostral brain dopamine and serum urea levels in individual animals in 10 *Gdnf^wt/wt^* and 12 *Gdnf^wt/hyper^* animals at P7.5 using the Correlation function in Microsoft Excel.

| **Dopamine levels in rostral brain, ng/g wet tissue** | **Genotype** | **Serum urea, mg/dL** |
| --- | --- | --- |
| 273.2 | wt/wt | 78.3 |
| 265.9 | wt/wt | 68.8 |
| 291.4 | wt/wt | 92.4 |
| 275.3 | wt/wt | 72.9 |
| 329.6 | wt/wt | 69 |
| Na | wt/wt | 73.2 |
| 254.5 | wt/wt | 103.4 |
| 203.3 | wt/wt | 107.6 |
| 239.5 | wt/wt | 107.6 |
| 284.9 | wt/wt | 73.9 |
| **Correlation function=-0.037** | | |
| 282.1 | wt/hyper | 197.7 |
| 167.8 | wt/hyper | 88.4 |
| 291.4 | wt/hyper | 90.2 |
| 318.9 | wt/hyper | 96.8 |
| 269.1 | wt/hyper | 104.2 |
| 299.7 | wt/hyper | 234.2 |
| 194.5 | wt/hyper | 266.9 |
| 244.4 | wt/hyper | 259.5 |
| 297.8 | wt/hyper | 208.9 |
| 278.1 | wt/hyper | 114.2 |
| 395.8 | wt/hyper | 110.4 |
| 420.9 | wt/hyper | 224.6 |
| **Correlation function=-0.73** | | |
