# Supplementary material for: GDNF Overexpression from the Native Locus Reveals its Role in the Nigrostriatal Dopaminergic System Function
Source: PLoS Genet. 2015 Dec 17;11(12):e1005710. doi: 10.1371/journal.pgen.1005710 (PMC4682981; doi:10.1371/journal.pgen.1005710)
Supplement: S4 Table — Levels of mature miRs expressed as fold difference relative to sno202 in adult mouse brain, developing mouse kidney and in HEK293 cells. N = 2 experiments. dSTR, dorsal striatum, E, embryonic day; Hs, Homo sapiens; Mm, Mus musculus; P, postnatal day; w, weeks. (DOCX) [file pgen.1005710.s005.docx]

**Supporting Table 4. miRNA expression levels in different tissues and cell lines.**

Levels of mature miRs expressed as fold difference relative to sno202 in adult mouse brain, developing mouse kidney and in HEK293 cells. N=2 experiments. dSTR, dorsal striatum, E, embryonic day; Hs, *Homo sapiens*; Mm, *Mus musculus*; P, postnatal day; w, weeks

| **Tissue/**  **cell** | **sno-202** | **miR-9** | **miR-30a** | **miR-30b** | **miR-33a** | **miR-96** | **miR-125a-5p** | **miR-125b-5p** | **miR-129** | **miR-133a** | **miR-133b** | **miR-146a** | **miR-182** | **miR-204** |
| --- | --- | --- | --- | --- | --- | --- | --- | --- | --- | --- | --- | --- | --- | --- |
| **Mm, rostral brain P7.5** | 1 | 5.14 ± 0.08 | 0.28 ± 0.02 | 0.67 ± 0.05 | 0.059 ± 0.025 | 0.0021 ± 0.0021 | 0.23 ± 0.03 | 6.52 ± 1.53 | 0.023 ± 0.005 | 0.0024 ± 0.0001 | 0.0019 ± 0.0002 | 0.011 ± 0.004 | 0.021 ± 0.020 | 0.16 ± 0.01 |
| **Mm, dSTR 10w** | 1 | 0.97 ± 0.14 | 0.014 ± 0.006 | 0.16± 0.08 | ne | ne | 1.46 ± 0.33 | 0.088 ± 0.060 | ne | 0.067 ± 0.020 | ne | 0.067 ± 0.006 | ne | 0.35 ± 0.23 |
| **Mm, kidney E11.5** | 1 | 0.0006 ± 0.0001 | 0.033 ± 0.001 | 0.24 ± 0.01 | 0.047 ± 0.004 | ne | 0.094 ± 0.001 | 0.31 ± 0.01 | ne | 0.0017 ± 0.0001 | 0.0021 ± 0.0001 | 0.0022 ± 0.0001 | 0.017 ± 0.001 | 0.0027 ± 0.0001 |
| **Hs, HEK293** | 1 | 4.42 ± 0.37 | ne | 37.3 ± 5.1 | 167.9±9.9 | 0.018 ± 0.014 | 17.34 ± 0.85 | 6.6 ± 0.6 | ne | 0.27 ± 0.02 | 0.081 ± 0.007 | 0.29 ± 0.02 | 1.70 ± 0.07 | 0.17 ± 0.02 |
