# Supplementary figures and images for: GDNF Overexpression from the Native Locus Reveals its Role in the Nigrostriatal Dopaminergic System Function
Source: PLoS Genet. 2015 Dec 17;11(12):e1005710. doi: 10.1371/journal.pgen.1005710 (PMC4682981; doi:10.1371/journal.pgen.1005710)

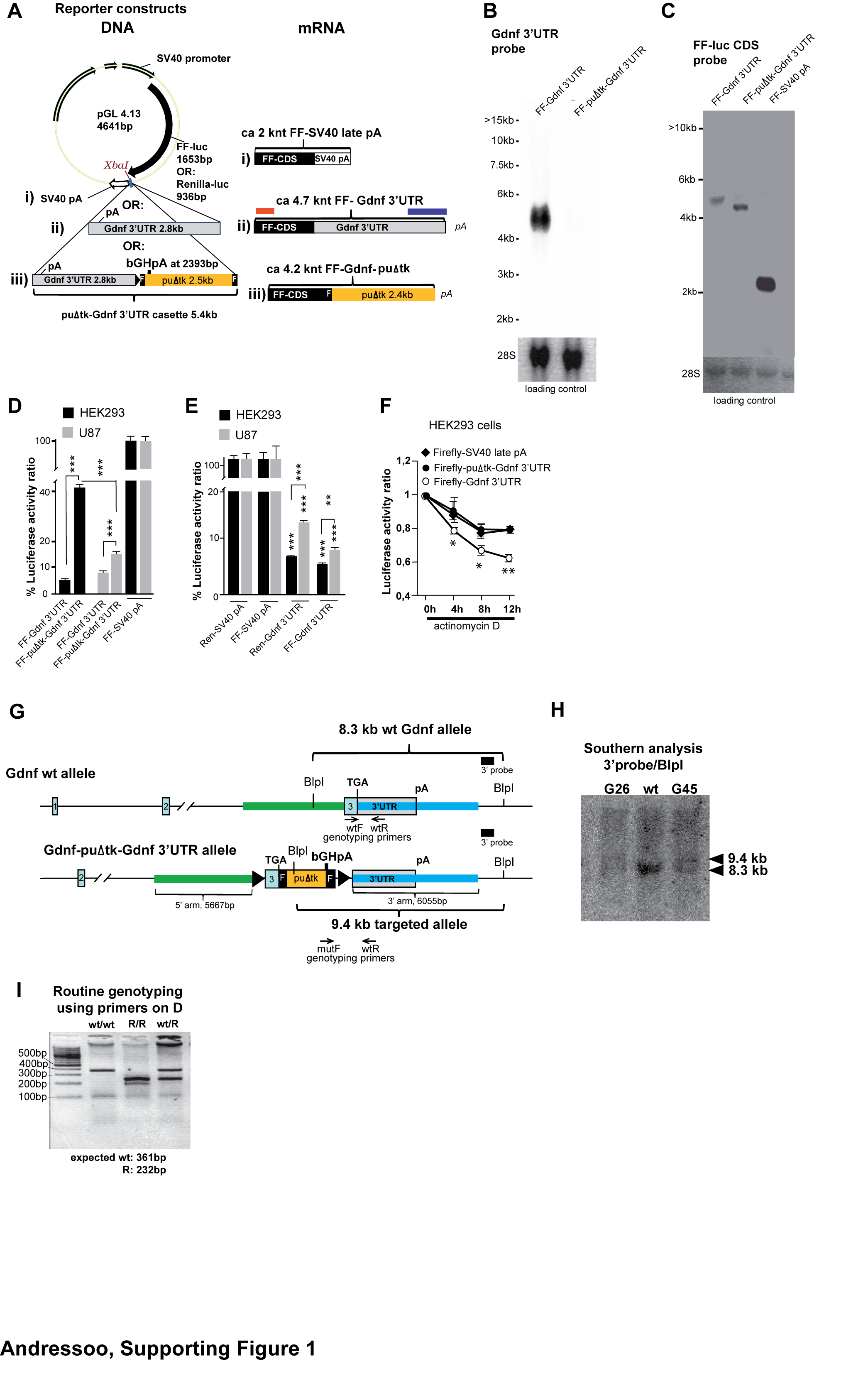

Supplement: S1 Fig — (A) Schematic representation of the reporter constructs and the derived mRNAs used in this study. Red and blue bar indicate probes used in the Northern blot for firefly luciferase CDS and Gdnf 3’UTR, respectively. (B) The puΔtk cassette blocks transcription to the Gdnf 3’UTR, measured by Northern blot analysis of expression from a construct harboring the Firefly (FF) coding sequence upstream of the puΔtk-Gdnf 3’UTR cassette in HEK293 cells; loading control: 28S ribosomal RNA. (C) Northern blot analysis of expression from construct harboring FF coding sequence proceeded with puΔtk-Gdnf 3’UTR cassette (S1 Fig A) in HEK293 cells (S1 Fig D); loading control: 28S ribosomal RNA. (D) Expression from equimolar amounts of the FF-Gdnf 3’UTR, FF-puΔtk-Gdnf 3’UTR and FF-SV40 pA constructs in U87 and HEK293 cells; FF-firefly luciferase; N = 3 experiments/construct with 3 replicates/experiment. (E) Gdnf 3’UTR reduces expression of both Renilla luciferase (Ren) and FF in HEK293 and U87 cells; N = 3 experiments/construct with 3 replicates/experiment (F) FF expression from constructs containing SV40 pA, puΔtk-Gdnf 3’UTR cassette and Gdnf 3’UTR in HEK293 cells after treatment with actinomycin D. Note that prevention of transcription of the native Gdnf 3’UTR by preceding puΔtk alleviates the post-transcriptional inhibition of the reporter. Renilla luciferase expression from a separate plasmid was used for normalization. N = 3 experiments/construct with 3 replicates/experiment. (G) Targeting strategy of the Gdnf locus. 5667 bp 5’ (green bar) and 6055 bp 3’ (blue bar) homologous arms, and Gdnf exon 3 until and including the stop codon were amplified with PCR from Gdnf-containing PAC and cloned into PmeI, NotI and HindIII sites in pFlexible [31], respectively, to generate Gdnf targeted allele. Sequence lengths are not drawn to scale. Arrows indicate primers used for routine genotyping; black bar indicates probe used for Southern blotting using BlpI restriction enzyme shown on (H). (I) Re [file pgen.1005710.s006.tif]

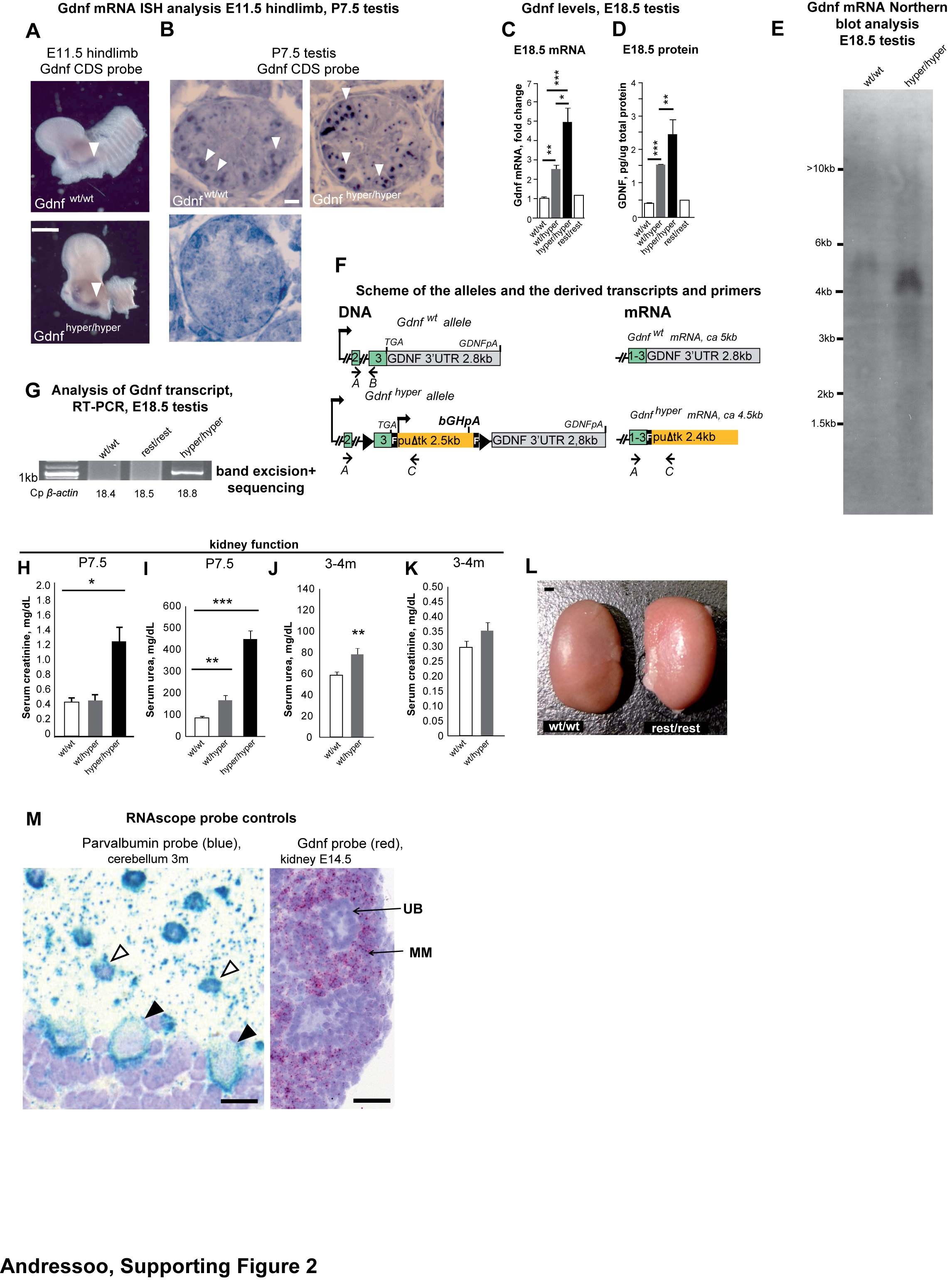

Supplement: S2 Fig — (A-B) Representative images of in situ hybridization of Gdnf mRNA using a probe against the CDS (stained blue, indicated with white arrow heads) in mice in whole-mount preparations of E11.5 hindlimb (A) and in paraffin sections from P7.5 testis (B). Note that Gdnf expression sites are comparable between genotypes, whereas the signal appears stronger in Gdnf hyper/hyper mice. N = 4 mice/group in A and N = 2 in B. (C-D) Gdnf mRNA (C) and protein (D) expression in the testis at E18.5 measured with QPCR and ELISA, respectively, shows allele dose-dependent increase in GDNF levels in GDNF hypermorphic mice. Note that GDNF protein levels in Gdnf 3’UTR rest/rest mice are normal (D). N = 2–5 mice/group in 2–3 experiments with 2–3 replicates/experiment (E) Representative image of Northern blot analysis of Gdnf mRNA in E18.5 testis using Gdnf CDS probe. Note that the size of the band is ca 500 bp shorter in Gdnf hyper/hyper mice compared to the Gdnf wt/wt mice, consistent with the size of transcripts derived from Gdnf-puΔtk fusion transcript (S1A–S1C Fig; and see S2 Fig F, G). N = 2 mice/group. (F) Schematic representation of the puΔtk-Gdnf 3’UTR (Gdnf hyper) allele for Gdnf, A and B designate primers used in QPCR analysis of Gdnf mRNA levels (C); primers A and C depict primers used for Gdnf transcript sequence analysis in (G). (G) Representative image of RT-PCR analysis from E18.5 testis using primers A and C as depicted in (F). The observed PCR product in Gdnf hyper/hyper mice was of expected length (977 bp) and it was sequenced for validation; N = 3 mice; beta-actin was used to verify cDNA quality. (H-I) Kidneys of P7.5 Gdnf hyper/hyper mice function poorly, as indicated by serum creatinine (H) and serum urea (I) levels. H, N = 3–12; I, N = 7–26 mice/group. (J-K) The blood serum of adult Gdnf wt/hyper mice contains slightly higher levels of urea (J), but not creatinine (K) compared to wild type mice. Note that two-fold variation in serum urea levels is considered normal in [file pgen.1005710.s007.tif]

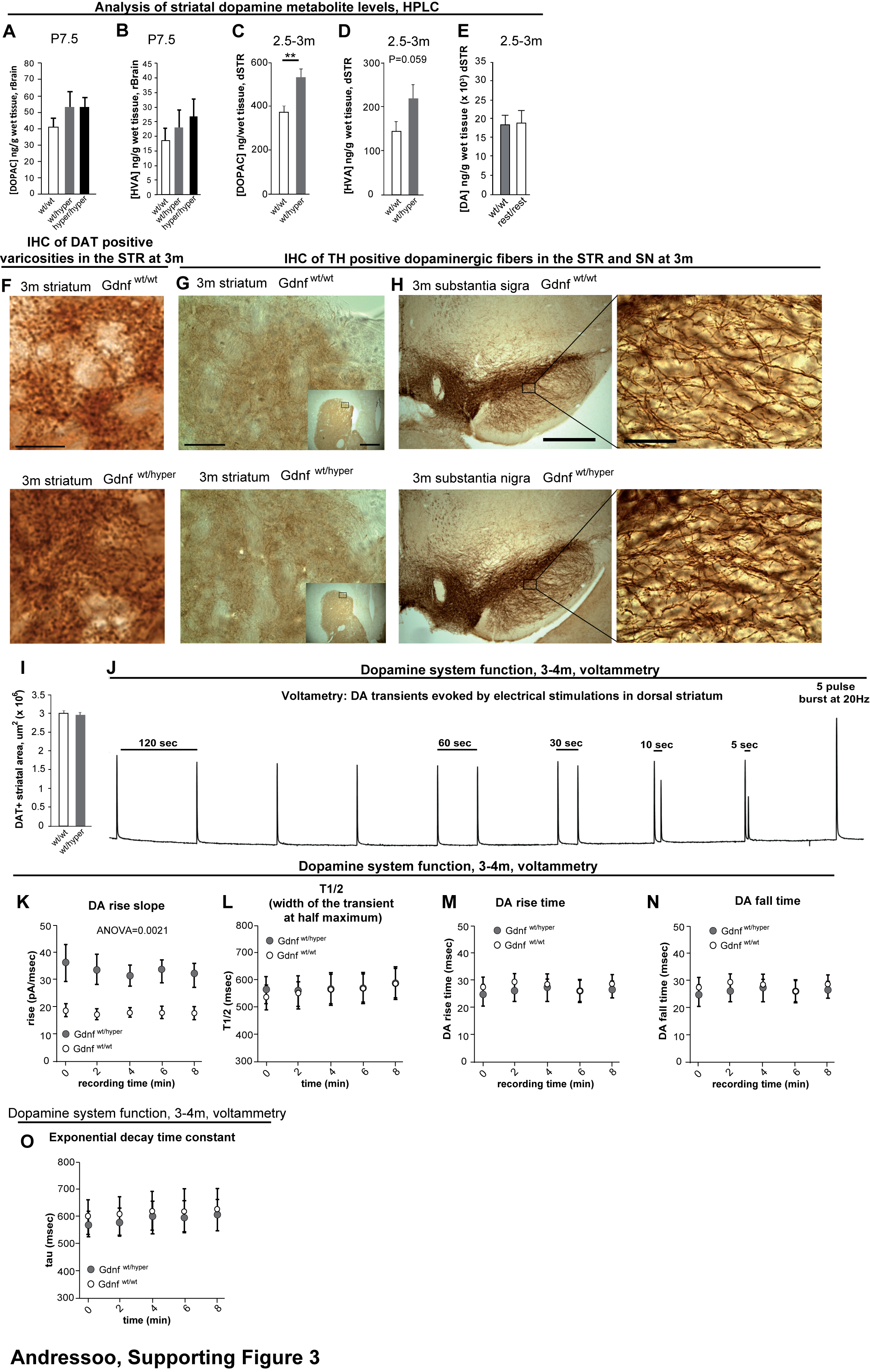

Supplement: S3 Fig — (A-B) HPLC analysis of DA metabolites DOPAC (A) and HVA (B) in P7.5 rostral brain. N = 5–8 mice/group. (C-D) HPLC analysis of DA metabolites DOPAC (C) and HVA (D) in the dSTR at 2.5–3 months of age. N = 11 for Gdnf wt/wt, 8 for Gdnf wt/hyper; P = 0.00521 for DOPAC and P = 0.0569 for HVA. (E) HPLC analysis of DA levels in the dSTR of Gdnf 3’UTR rest/rest and Gdnf wt/wt mice; N = 6 mice/group. (F) Representative images of DAT immunostaining in the dSTR, N = 7–9 mice/group. (G-H) Representative images of TH immunostaining in the dSTR (G) and midbrain (H). In total, 8 mice were analyzed from each genotype. (I) Average striatal area size measured from DAT+ sections. (N = 9 for Gdnf wt/wt, 7 for Gdnf wt/hyper (J) Representative recording trace of DA events stimulated by single electrical pulses at 2 min intervals, by paired stimulations at shown intervals, and by a burst of 5 stimuli at 20 Hz. (K-O) Rise and decay parameters of the first five DA events evoked by single-pulses. N = 5–7 mice/group with 1–3 striatal slices/mouse analyzed. (K) DA rise slope is steeper in the striata of Gdnf wt/hyper mice [two-way repeated measures ANOVA, F (1,29) = 11.47, P = 0.0021], indicating that more dopamine is released from the terminals during the rise phase. (L) T ½ times, i.e., the width of the event at half of the maximum. (M) Rise time, i.e. the time for DA events to rise from baseline to the maximum. (N) The fall time of the DA events. (O) The exponential decay time constant of the DA transients. Abbreviations: DA, dopamine; DOPAC, 3,4-dihydroxyphenylacetic acid; dSTR, dorsal striatum; HPLC, high performance liquid chromatography; HVA, homovanillic acid; IHC, immunohistochemistry; m, months; P, postnatal day. Scale bars: F, 10 μm; G, 75 μm; in inset 1 mm; H, 0.5 mm; in inset 30 μm. (TIF) [file pgen.1005710.s008.tif]

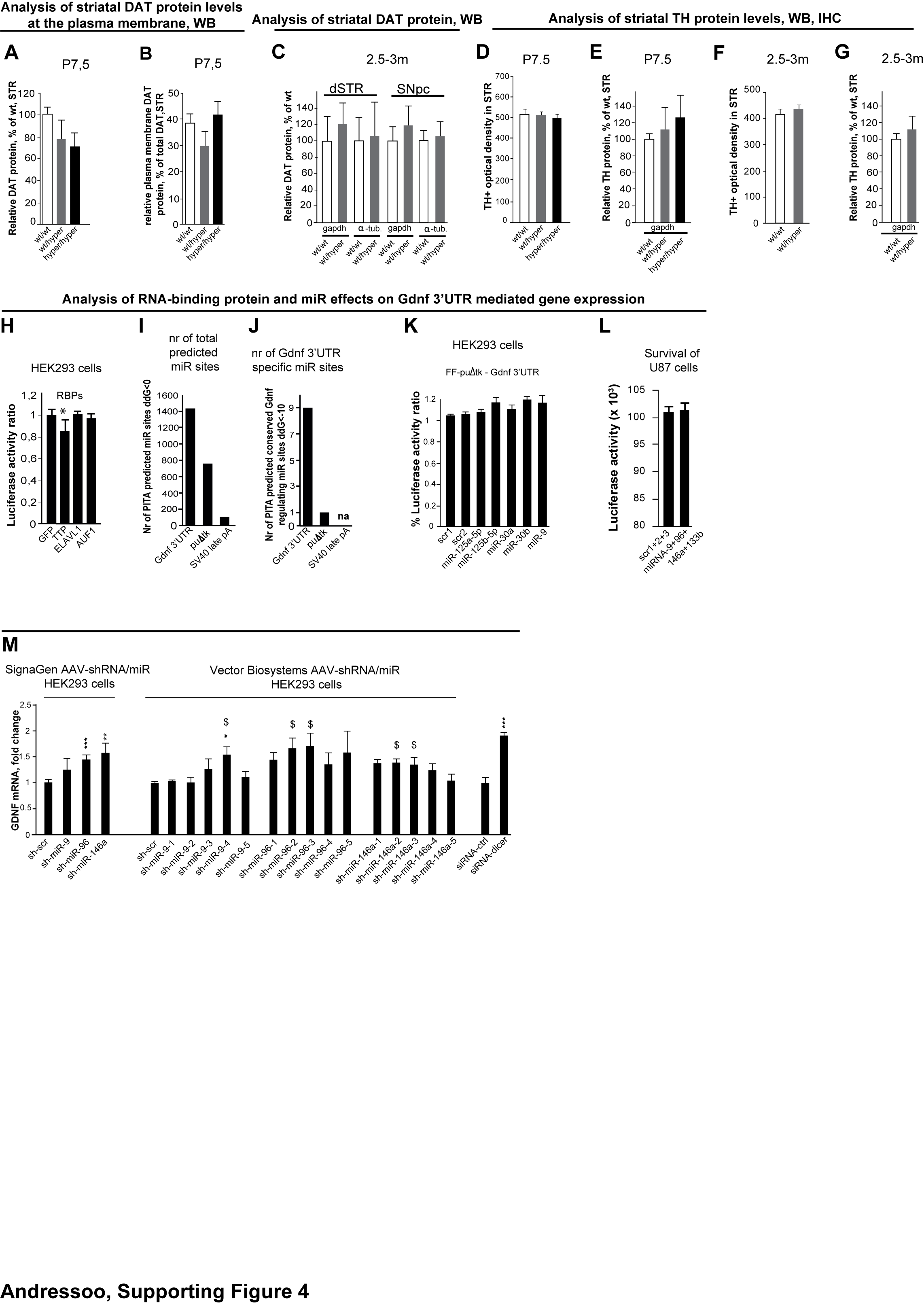

Supplement: S4 Fig — (A-B). Total DAT protein levels (A) and plasma membrane associated DAT levels (B) at P7.5, measured by western blotting. N = 4 mice/group. (C) DAT protein levels in the dSTR and SNpc at 2.5-3m, measured by western blotting. Gapdh or α-tubulin was used to normalize loading, as indicated in the figure. N = 5 mice/group. (D) OD of striatal TH+ fibers at P7.5, reflecting striatal TH levels and the density of striatal dopaminergic innervation at the macroscopic level. N = 7 mice/group. (E) TH protein levels in the dSTR at P7.5, measured by western blotting. Gapdh was used to normalize loading. N = 4 mice/group. (F) OD of striatal TH+ fibers at 2.5–3 months of age. N = 10–11 mice/group. (G) TH protein levels in the dSTR at 2.5–3 months of age, measured by western blotting. Gapdh was used to normalize loading. N = 5 mice/group. (H) Luciferase expression from Firefly-Gdnf 3’UTR construct in HEK293 cells after co-transfection with constructs encoding RBPs. Renilla luciferase expression from a separate plasmid was used for normalization and GFP was used as a negative control. N = 3 experiments with 3 biological repeats each. (I) The number of predicted miR binding sites (ddG<0) in Gdnf 3’UTR, puΔtk and SV40 late pA signal, analyzed with PITA software [4]. (J) The number of predicted strong binding sites for Gdnf-specific miRs (ddG<-10) in Gdnf 3’UTR, puΔtk and SV40 late pA signal, analyzed with PITA software [4]. Selection of Gdnf-specific miRs was based on TargetScan analysis using the most stringent conditions. (K) Luciferase expression from Firefly-puΔtk-Gdnf 3’UTR construct in HEK293 cells after co-transfection with Gdnf-regulating pre-miRs. Note that miRs that inhibit luciferase expression via the native Gdnf 3’UTR (Fig 5B) do not suppress the expression from reporter construct containing the Firefly-puΔtk-Gdnf 3’UTR cassette. N = 3 experiments with 3–5 biological repeats. (L) Luciferase activity in an ATP-based survival assay in U87 cells after co-transfection with pre- [file pgen.1005710.s009.tif]
